# Ion channel function of polycystin‐2/polycystin‐1 heteromer revealed by structure‐guided mutagenesis

**DOI:** 10.1002/1873-3468.70059

**Published:** 2025-05-12

**Authors:** Tobias Staudner, Juthamas Khamseekaew, M. Gregor Madej, Linda Geiges, Bardha Azemi, Christine Ziegler, Christoph Korbmacher, Alexandr V. Ilyaskin

**Affiliations:** ^1^ Institute of Cellular and Molecular Physiology, Friedrich‐Alexander‐Universität Erlangen‐Nürnberg Erlangen Germany; ^2^ Department of Biophysics II/Structural Biology University of Regensburg Germany; ^3^ Present address: Department of Physiology Faculty of Medicine, Khon Kaen University Khon Kaen Thailand

**Keywords:** ADPKD, electrophysiology, polycystin‐1, polycystin‐2, TRP channels

## Abstract

Mutations in polycystin‐1 (PC1) or polycystin‐2 (PC2) cause autosomal‐dominant polycystic kidney disease (ADPKD). Structural data suggest that one PC1 and three PC2 form heterotetrameric ion channels with an ion permeation pathway blocked by PC1 (R4100, R4107, and H4111) and PC2 (L677, N681) residues. Here, we demonstrate that replacing these residues with alanines results in a gain‐of‐function (GOF) PC2/PC1 construct with distinct selectivity properties compared to PC2 homomers. We also show preferential formation of PC2/PC1 heteromeric complexes over PC2 homomers. Re‐interpretation of published PC2/PC1 cryo‐electron microscopy data, combined with cysteine modification experiments, suggests that the pore‐forming domain of PC1 adopts a canonical TRP channel‐like conformation. This novel PC2/PC1 GOF construct offers the opportunity to investigate the functional impact of ADPKD mutations.

## Graphical abstract

Mutations in polycystin‐1 (PC1) or polycystin‐2 (PC2) cause autosomal‐dominant polycystic kidney disease (ADPKD). We generated a novel gain‐of‐function PC2/PC1 heteromeric ion channel by mutating pore‐blocking residues. Moreover, we demonstrated that PC2 will preferentially assemble with PC1 to form heteromeric complexes when PC1 is co‐expressed. Furthermore, we provided evidence for a canonical TRP channel‐like conformation of the PC1 pore loop.

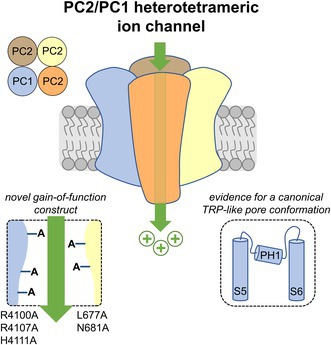

## Abbreviations

**ADPKD**, Autosomal‐dominant polycystic kidney disease

**PC1**, polycystin‐1

**PC2**, polycystin‐2

**GOF**, gain‐of‐function

**ESRD**, end‐stage renal disease

**TRP**, transient receptor potential

**cryo‐EM**, cryo‐electron microscopy

**ER**, endoplasmic reticulum

**GPCR**, G protein‐coupled receptor

**GPS**, GPCR proteolysis site

**GAIN**, GPCR autoproteolysis‐inducing

**NTF**, N‐terminal fragment

**CTF**, C‐terminal fragment

**NMDG**, N‐methyl‐D‐glutamine

**WT**, wild‐type

**DMA**, Dimethylamin

**DEA**, Diethylamin

**cRNA**, complementary RNA

**PH**, pore helix

**MTSET**, 2‐(Trimethylammonium)ethyl methanethiosulfonate

**TEVC**, two‐electrode voltage‐clamp

Autosomal‐dominant polycystic kidney disease (ADPKD) is the most common hereditary kidney disease and is responsible for 5–10% of all cases of end‐stage renal disease (ESRD) [1]. Its main manifestation is the development and progressive growth of multiple fluid‐filled renal cysts which compromise kidney function [2]. In the majority of cases, ADPKD is caused by mutations in the PKD1 or PKD2 gene [3, 4] coding for polycystin‐1 (PC1) and polycystin‐2 (PC2), respectively [5, 6]. On a cellular level, ADPKD onset is most likely triggered by a two‐hit mechanism requiring one germline mutation (“first hit”) and a second somatic mutation (“second hit”) [7, 8, 9, 10, 11]. Despite intensive research efforts, the physiological function of PC1 and PC2 and their mechanistic role in ADPKD pathogenesis remain unclear.

PC2 is a member of the transient receptor potential (TRP) family of ion channels [12] bearing its hallmark features of 6 transmembrane domains (S1–S6), intracellular N‐ and C termini, and a putative ion permeation pathway between the fifth (S5) and the sixth (S6) transmembrane domain [13]. Recently published cryo‐electron microscopy (cryo‐EM) structures of PC2 indicate that the protein can form homotetrameric ion channels [14, 15, 16, 17, 18]. Therefore, homotetrameric PC2 probably functions as a nonselective cation channel like most TRP channels [12, 19]. However, both the (patho‐)physiological role and the electrophysiological characteristics of PC2 remain controversial in the literature [20, 21]. Early studies identified the endoplasmic reticulum as one of the main sites of PC2 expression [22, 23]. Therefore, it was proposed that PC2 either mediates Ca^2+^ release from the endoplasmic reticulum (ER) [23, 24] or interacts with ryanodine receptors [25] and IP_3_ receptors [26, 27]. A more recent study proposed that PC2 might function as a K^+^ ion channel in the ER facilitating K^+^‐Ca^2+^ counterion exchange, thereby promoting IP_3_‐mediated Ca^2+^ release from ER [28]. Another cellular location of PC2 is the primary cilium [29, 30]. Primary cilia malfunction is thought to contribute to ADPKD pathogenesis [31, 32, 33]. Importantly, it has been reported that PC2 forms active ion channels in primary cilia, which can be potentiated by depolarization and intraciliary Ca^2+^ [34, 35]. Functional characterization of PC2 in heterologous expression systems remains challenging because the channel produces very low baseline currents [36, 37, 38]. In recent years, several gain‐of‐function (GOF) mutations of PC2 have been generated [36, 39, 40]. This facilitated the electrophysiological characterization of PC2 in heterologous expression systems and made it possible to study the effects of ADPKD‐associated mutations on PC2 ion channel function [38, 41]. Furthermore, binding sites for small‐molecule modulators of a GOF PC2 mutant have recently been characterized [18]. Despite these advances, however, no specific pharmacological activators or inhibitors of native PC2 have been identified to date.

PC1 is a 4303 residue‐long transmembrane protein consisting of 11 transmembrane segments, a large ~3000 residue‐long extracellular N terminus, and a short intracellular C terminus [5, 42]. The N terminus of PC1 undergoes autocatalytic cleavage between L3048 and T3049 at the G protein‐coupled receptor (GPCR) proteolysis site (GPS) of its GPCR autoproteolysis‐inducing (GAIN) domain [43, 44]. This produces the N‐terminal (PC1‐NTF) and the C‐terminal fragment (PC1‐CTF), which remain noncovalently bound to each other. Because of these properties, PC1 resembles adhesion GPCRs [45, 46]. Furthermore, a putative tethered peptide agonist (“Stachel” domain) in the N‐terminal portion of PC1‐CTF [47, 48] and a G‐protein activation sequence in the intracellular C‐terminus of PC1 have been described [49, 50, 51]. According to the generally accepted paradigm, PC1 and PC2 functionally interact via their C‐terminal coiled‐coil domains [52, 53, 54, 55] and are colocalized in primary cilia [29]. This localization suggests an important yet unknown chemo‐ or mechanosensory function of the PC2/PC1 complex [4]. However, it was demonstrated that PC1 is not required for basal ion channel activity of PC2 in primary cilia [35]. Moreover, conflicting findings regarding the effect of PC1 on PC2 ion channel function in heterologous expression systems have been reported [40, 56, 57, 58]. Therefore, it is still a matter of debate whether the PC2/PC1 complex functions as an ion channel under physiological conditions and whether its disturbed ion channel function contributes to the onset and progression of ADPKD.

Recent cryo‐EM data intriguingly indicate that PC1 can replace one PC2 subunit to form a heteromeric PC2/PC1 ion channel with a 3 : 1 stoichiometry [59]. Moreover, co‐expression of PC1 has been shown to alter ion channel properties of a gain‐of‐function PC2 construct, suggesting that PC1 contributes to the channel pore [40]. These findings suggest that PC2 may contribute to the formation of two different ion channels—PC2 homomers and PC2/PC1 heteromers—each with distinct permeability characteristics [60]. In this study, we used structure‐guided mutagenesis to provide further evidence for PC2/PC1 function as a heteromeric ion channel. We generated a novel GOF PC2/PC1‐CTF ion channel and characterized its properties. Furthermore, we demonstrated that PC2 preferentially associates with PC1‐CTF to form heteromeric complexes, likely with a 3 : 1 stoichiometry consistent with the cryo‐EM data. The initial interpretation of the cryo‐EM densities suggested that PC1 has a noncanonical architecture in its pore‐forming domains, where the S6 helix consists of two separate segments, S6a and S6b, with the proximal segment S6a resembling the pore helix 1 [59]. However, our re‐interpretation of these cryo‐EM data did not confirm this structural feature. Instead, our revised model suggests that the pore‐forming domain of PC1 has a canonical TRP‐like conformation. Combined with our functional data, this supports the conclusion that PC1‐CTF directly contributes to the channel pore of the PC2/PC1‐CTF heteromeric ion channel.

## Materials and methods

### Reagents and tools

See Supporting Information Table S1.

### cDNA constructs

Full‐length complementary DNAs (cDNAs) encoding human polycystin‐1 and polycystin‐2 were kindly provided by R. Witzgall (Regensburg, Germany). The first 3048 amino acids of PC1 were removed to obtain the C‐terminal fragment of PC1 (PC1‐CTF) using Gibson assembly. For heterologous expression in *Xenopus laevis* oocytes, PC1‐CTF and PC2 were subcloned into the pTLN vector [61]. Mutations were introduced to PC1‐CTF and PC2 using the QuikChange Lightning site‐directed mutagenesis kit (Agilent, Santa Clara, CA, USA). For immunoblotting, PC1‐CTF and PC2 constructs were modified by attaching a C‐terminal V5‐tag (GKPIPNPLLGLDST) and an N‐terminal HA‐tag (YPYDVPDYA), respectively. In addition, an Ig k‐chain leader sequence (METDTLLLWVLLLWVPGSTGD) was introduced to the N terminus of PC1‐CTF to improve its cell surface expression [40]. Sequences of all constructs were routinely verified by a commercially available sequence analysis service (LGC Genomics GmbH, Germany). Plasmids were linearized using the MluI restriction enzyme and used as templates for cRNA synthesis using SP6 RNA polymerase (mMessage mMachine SP6, Invitrogen by Thermo Fisher Scientific, Waltham, MA, USA).

### Isolation of *Xenopus laevis* oocytes and two‐electrode voltage‐clamp (TEVC) experiments

Isolation of *Xenopus laevis* oocytes and two‐electrode voltage‐clamp (TEVC) experiments were performed essentially as described previously [38, 62]. In brief, ovarian lobes were excised by partial ovariectomy under anesthesia with Tricain 0.2%, in accordance with the principles of German legislation, with approval by the animal welfare officer for the University of Erlangen‐Nürnberg (FAU), and under the governance of the state veterinary health inspectorate. The isolated sections of the ovarian lobes were then treated with 600–700 U·mL^−1^ collagenase type‐2 from *Clostridium hystolyticum* (Sigma‐Aldrich, St. Louis, MO, USA) for 3–4 h at 19 °C. Subsequently, stage V‐VI oocytes were injected with the amount of cRNA coding for the respective PC2 and/or PC1‐CTF construct as indicated in the figures or the figure legends. To avoid background currents mediated by endogenous connexin 38 hemichannels under divalent cation‐free conditions, 3 ng of an antisense phosphorothioate DNA oligomer corresponding to nucleotides −5 to +25 relative to the coding region of connexin 38 (5′‐GCTTTAGTAATTCCCATCCTGCCATGTTTC‐3′; AS Cx38) was co‐injected in every oocyte. Oocytes injected only with the AS Cx38 were used as the control group. Following the cRNA injection, oocytes were incubated at 19 °C for 48–72 h in ND9: NaCl 9 mm, KCl 2 mm, N‐methyl‐d‐glutamine‐Cl (NMDG‐Cl) 87 mm, CaCl_2_ 1.8 mm, MgCl_2_ 1 mm, HEPES 5 mm, pH 7.4 with Tris supplemented with 100 units·mL^−1^ of sodium penicillin and 100 μg·mL^−1^ of streptomycin sulfate.

In TEVC experiments conducted at room temperature, oocytes were continuously superfused with a bath solution as indicated in the figures. The standard NaCl bath solution was composed as follows: NaCl 96 mm, KCl 4 mm, CaCl_2_ 1 mm, MgCl_2_ 1 mm, HEPES 10 mm, pH = 7.4 adjusted with Tris. CaCl_2_ and MgCl_2_ were excluded from the standard NaCl bath solution to create a NaCl bath solution nominally free of divalent cations (NaCl øMg^2+^ øCa^2+^). In the latter solution, 95 mm NaCl was replaced with the same concentration of NMDG‐Cl to obtain NMDG‐Cl divalent cation‐free bath solution (NMDG‐Cl øMg^2+^ øCa^2+^). Gravity‐based bath solution exchanges were controlled by a magnetic valve system (ALA VM8; ALA Scientific Instruments, Farmingdale, NY, USA). The holding potential was constantly kept at −60 mV. At the end of each solution application, a voltage step protocol with consecutive 1000 ms voltage steps in 20 mV increments starting with a hyperpolarizing pulse to 100 mV from a holding potential of −60 mV and ending with a depolarizing pulse to +40 mV was performed. The current values measured during the final 300 ms of each pulse were used to construct the corresponding I/V‐plot.

To estimate the channels' selectivity for small inorganic monovalent cations (Na^+^, Li^+^, K^+^) and mid‐sized organic monovalent cations (DMA^+^, DEA^+^) (Fig. 3A–D; Figs S9A,B, S10), bath solutions with the following compositions were used: NaCl, LiCl, KCl, DMA‐Cl, DEA‐Cl, or NMDG‐Cl 100 mm, HEPES 10 mm, pH 7.4 adjusted with Tris. To correct for endogenous oocyte conductances, average whole‐cell current values obtained in the corresponding bath solution in control oocytes (Fig. S9) were subtracted from current values obtained in polycystin‐expressing oocytes from the same batch. The permeability ratios for monovalent cations (P_Na_: P_K_: P_Li_: P_DEA_: P_DMA_: P_NMDG_) were calculated from the corresponding reversal potential shifts using the modified GHK equation [63]:

(1) $${P_{X}}^{+}/{P_{\mathrm{Na}}}^{+}=e^{\text{ΔErev}F/\mathrm{RT}}$$

where ΔE_rev_ represents the shift of the reversal potential (E_rev_) caused by replacing Na^+^ in the bath solution with cation X^+^ [$\Delta E_{\mathrm{rev}}={E_{\mathrm{rev},X}}^{+}-{E_{\mathrm{rev},\mathrm{Na}}}^{+}$], *F* is Faraday's constant, *R* is the universal gas constant, and *T* is the absolute temperature in Kelvin.

To estimate the channels' Ca^2+^ permeability, a bath solution with a high Ca^2+^ concentration (CaCl_2_ 50 mm, HEPES 10 mm, pH 7.4 adjusted with Tris) was applied for 60 s to oocytes continuously clamped at the standard holding potential of −60 mV (Fig. 3E,F). Current–voltage (I/V) relationships were obtained using a 2‐s voltage ramp protocol from −100 mV to +40 mV performed at the end of the CaCl_2_ application period. Bath solutions containing 50 mm BaCl_2_ or 50 mm MgCl_2_ instead of 50 mm CaCl_2_ were used to assess the channels' permeability for Ba^2+^ or Mg^2+^, respectively. Data analysis was performed using the programs Microsoft Excel and “Nest‐o‐Patch” (http://sourceforge.net/projects/nestopatch) written by Dr. V. Nesterov (Friedrich‐Alexander‐Universität Erlangen‐Nürnberg, Institute of Cellular and Molecular Physiology, Erlangen, Germany).

### Detection of polycystin‐1 and polycystin‐2 at the cell surface

Cell surface expression of PC1‐CTF and PC2 was assessed using a biotinylation approach essentially as described previously [38, 62, 64]. In brief, oocytes were incubated with EZ‐Link Sulfo‐NHS‐SS‐Biotin (Thermo Fisher Scientific) and lysed mechanically through a 27G‐needle. Subsequently, biotinylated proteins were separated from intracellular proteins with Pierce NeutrAvidin agarose beads (Thermo Fisher Scientific). After protein separation by electrophoresis, V5‐tagged PC1‐CTF or HA‐tagged PC2 was detected on a PVDF membrane. The primary antibodies used were a monoclonal rat anti‐HA antibody (Roche Diagnostics, Rotkreuz, Switzerland; dilution: 1:1000) and a monoclonal mouse anti‐V5 antibody (Thermo Fisher Scientific, dilution: 1:2000). The secondary antibodies used were horseradish peroxidase‐conjugated goat‐anti‐rat antibody (Jackson Immunoresearch, West Grove, PA, USA; dilution: 1:10 000) and horseradish peroxidase‐conjugated goat‐anti‐mouse (Abcam, Cambridge, UK; dilution: 1:50 000). To confirm the separation of cell surface proteins from intracellular proteins, western blots were subsequently stripped and reanalyzed using a polyclonal rabbit anti‐β‐actin antibody (Sigma‐Aldrich, dilution: 1:5000) and a secondary horseradish peroxidase‐conjugated goat‐anti‐rabbit antibody (Invitrogen by Thermo Fisher Scientific; dilution: 1:40 000).

### Co‐immunoprecipitation (co‐IP)

Thirty oocytes per group were washed three times in ND9 on ice. Oocytes were subsequently transferred into 1 mL of lysis buffer (Tris 25 mm, NaCl 150 mm, EDTA 1 mm, NP40 1%, Glycerol 5%, supplemented with Roche cOmplete™ protease inhibitor cocktail, pH = 7.4) and mechanically lysed using a syringe and 27G‐needle. Lysates were incubated by rotating at 4 °C for 1 h. Lysates were then centrifuged for 30 min at 10 000 ***g*** at 4 °C and supernatants were collected. Before application to the collected supernatants, anti‐HA (Thermo Fisher Scientific) or anti‐V5 (Sigma‐Aldrich) antibody‐coated magnetic beads were washed two times in TBST (Tris 25 mm, NaCl 150 mm, TWEEN 0.05%, pH = 7.5). 25 μL of the respective beads was pipetted on the collected supernatant and subsequently incubated for 30 min at room temperature by rotation. Thereafter, beads were collected and washed three times in TBST and once in nuclease‐free water for 5 min at 4 °C. For elution of the precipitated proteins, the beads were incubated for 10 min in 100 μL of elution buffer (0.1 m glycine, pH = 2) at room temperature. After removal of the magnetic beads, eluted protein fractions were neutralized with 15 μL of neutralization buffer (1 m Tris, pH = 8.5).

### Re‐analysis of cryo‐electron microscopy data and modeling approaches

The structure of the human PC2/PC1 heterotetrameric complex was retrieved from the RCSB protein database (PDB ID: 6A70; [59]) and the corresponding EM density was obtained from EMDB (Electron Microscopy Data Bank; EMD‐6991). To generate a homology model of the PC1 C‐terminal domain (CTD), sequence alignment between the template structure (PDB ID: 5MKF; [16]) and the target sequence of PC1 CTD was performed using the CLUSTAL Omega multiple sequence alignment tool available at www.uniprot.org/align. MODELLER [65] was then employed for comparative structure modeling to generate an initial model of the PC1 CTD, which was subsequently fitted into the corresponding EM density (EMD‐6991). Following manual corrections to the sequence alignment, the final model underwent iterative refinement using REFMAC [66] against the EM density. Regions that could not be modeled based on homology were either manually adjusted or built *de novo* using COOT [67].

### Statistical analysis and data presentation

N indicates the number of different batches of *Xenopus laevis* oocytes, and *n* indicates the number of individual oocytes analyzed per experimental group. Data are presented as mean ± SEM. Statistical evaluation was done using an appropriate statistical test as indicated in the figure legends using GraphPad Prism (GraphPad Software Inc.). The normal distribution of data was assessed using the D'Agostino‐Pearson omnibus test. Graphical representations were created using GraphPad Prism. Structure visualizations were prepared using UCSF Chimera developed by the Resource for Biocomputing, Visualization, and Informatics at the University of California, San Francisco, with support from the National Institutes of Health P41‐GM103311 [68].

## Results and discussion

### Wild‐type PC2 expressed alone or in combination with the C‐terminal fragment of PC1 (PC1‐CTF) did not reveal substantial ion channel activity in the oocyte expression system

To investigate the functional interaction between human PC2 and human PC1, we used *Xenopus laevis* oocytes as a heterologous expression system. Removal of the divalent cations (Ca^2+^ and Mg^2+^) from the bath solution was used as an established maneuver to reveal PC2 ion channel activity [36, 37, 38, 39]. In oocytes expressing PC2 WT, only marginal sodium inward currents could be detected under divalent cation‐free conditions (PC2; Fig. S1A,D). These inward currents were completely blocked when extracellular Na^+^ was replaced by the large organic cation NMDG^+^, to which PC2 is essentially impermeable. These experiments demonstrated that the Na^+^ conductance of oocytes expressing PC2 WT was very small, consistent with our previous findings [37, 38]. It has been reported that the C‐terminal fragment of PC1 (PC1‐CTF) is sufficient to study PC2/PC1 interaction in the oocyte expression system [40]. Co‐expression of PC2 with PC1‐CTF (amino acid residues 3049–4303) reduced inward currents elicited by Ca^2+^ and Mg^2+^ removal (PC2 + PC1‐CTF; Fig. S1B,D) to a level below that in oocytes expressing PC2 alone and only slightly higher than that in control oocytes (Fig. S1C,D). Importantly, decreased currents in this group of oocytes were not due to reduced PC2 expression at the cell surface. On the contrary, PC2 cell surface expression was increased in oocytes co‐expressing PC2 and PC1‐CTF compared to oocytes expressing PC2 alone (Figs S1E, S2). This latter finding is consistent with a previously described stimulatory effect of PC1 on PC2 cell surface expression [54, 55]. In summary, co‐expression of PC1‐CTF with PC2 reduced sodium inward currents in oocytes. In contrast, an earlier study in CHO cells reported that cells co‐expressing PC1 and PC2 showed higher currents than cells transfected with PC1 or PC2 alone [56].

### Alanine substitutions of the pore‐blocking residues in PC2 and PC1‐CTF produced a novel gain‐of‐function (GOF) PC2/PC1‐CTF construct

**Fig. 1:**
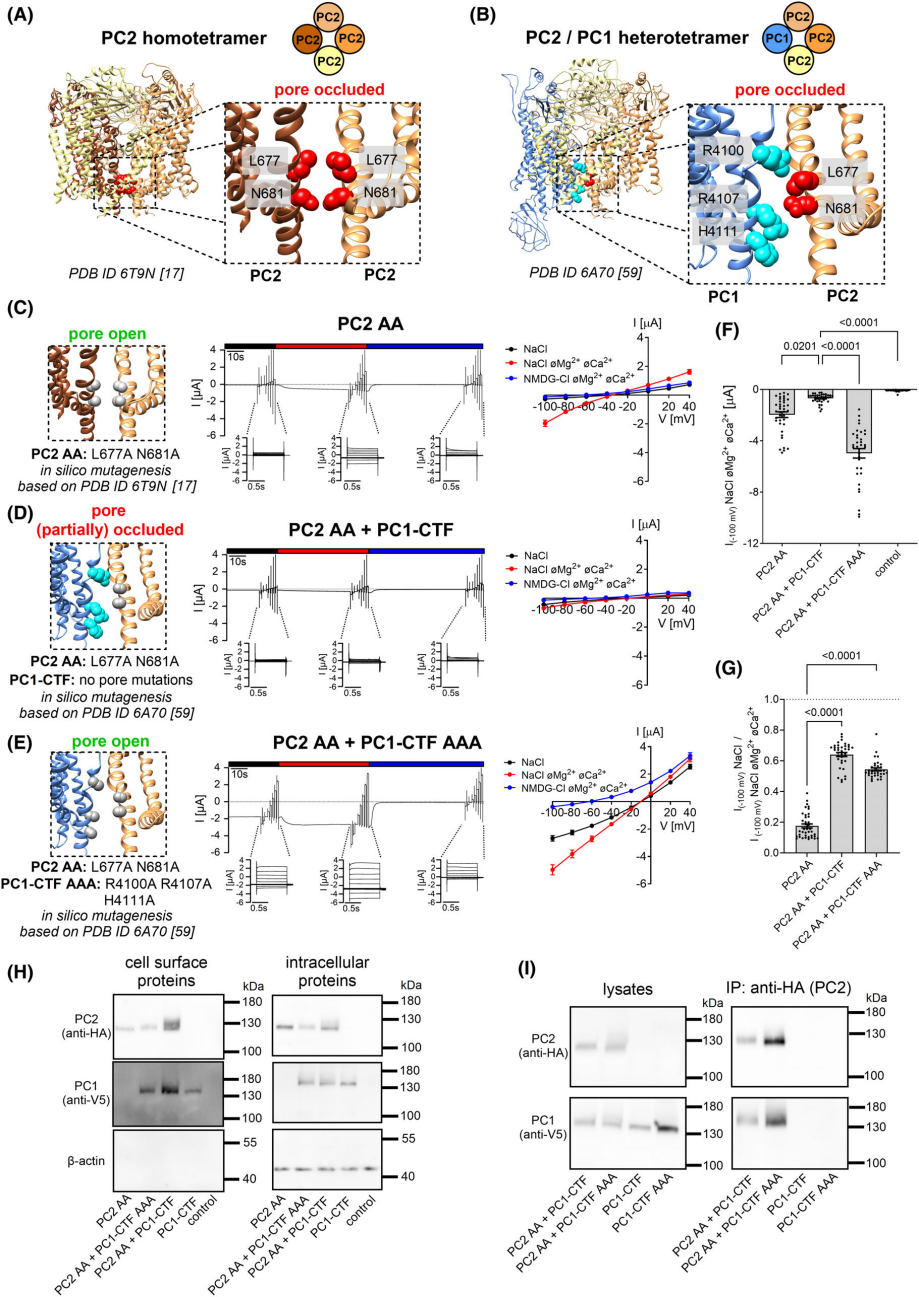
Generation of a gain‐of‐function (GOF) PC2/PC1‐CTF ion channel. (A, B) Side view of human homotetrameric PC2 (A) or heterotetrameric PC2/PC1 complex (B) in ribbon representation generated using atom coordinates from PDB entry 6T9N [17] or 6A70 [59], respectively. Individual protomers are colored according to the schematic diagram shown above the structure. The insets show a portion of PC2 or PC2/PC1 on an expanded scale. Pore‐blocking residues are labeled and colored in *red* for PC2 and in *cyan* for PC1 with atoms shown as spheres. (C–E) *Left panels* Localization of alanine substitutions in gray within the pore of homomeric PC2 (C) or heteromeric PC2/PC1 ion channels (D, E) introduced to create PC2 AA and PC1‐CTF AAA GOF constructs. *Middle panels* Representative whole‐cell current traces obtained in individual oocytes injected with 2.5 ng cRNA encoding human PC2 AA alone (C), or with additional co‐injection of 5 ng cRNA encoding PC1‐CTF (D) or PC1‐CTF AAA (E). Presence of standard NaCl bath solution with or without divalent cations (øMg^2+^øCa^2+^) or NMDG‐Cl bath solution without divalent cations is indicated by black, red, and blue bars, respectively. Overlays of whole‐cell current traces resulting from voltage step protocols are shown below the continuous current recordings. *Right panels* Average I/V‐plots (mean ± SEM) were constructed from similar recordings as shown in *middle panels* (C: *n* = 39, *N* = 4; D: *n* = 37, *N* = 4; E: *n* = 34, *N* = 4). *n* indicates the number of individual oocytes analyzed per experimental group and N indicates the number of different batches of oocytes. (F) Maximal currents in PC2 AA *vs*. PC2 AA/PC1‐CTF *vs*. PC2 AA/PC1‐CTF AAA expressing oocytes. The maximal inward currents reached during the application of hyperpolarizing pulses of −100 mV in divalent free NaCl (NaCl øMg^2+^øCa^2+^) bath solution are shown. Data are from the same experiments as summarized in the I/V plots (C–E) and from control experiments (control) using oocytes solely injected with AS Cx38 (*n* = 38, *N* = 4). The *P*‐values were calculated by the Kruskal–Wallis test with Dunn's *post hoc* test. (G) Summary of the relative inhibitory effects of Ca^2+^ and Mg^2+^ on PC2 AA‐, PC2 AA/PC1‐CTF‐ and PC2 AA/PC1‐CTF AAA‐mediated sodium inward currents (same experiments as in C–E). In each individual recording, the maximal inward current reached in NaCl bath solution with divalent cations at −100 mV was normalized to the maximal inward current measured in NaCl bath solution without divalent cations at −100 mV. The *P*‐values were calculated by the Kruskal‐Wallis test with Dunn's *post hoc* test. (H) Representative western blot analysis of cell surface (*left panels*) and intracellular (*right panels*) expression of PC2 and PC1‐CTF constructs in oocytes from one batch. Similar results were obtained in two other batches (*N* = 3; Fig. S3). (I) Validation of PC2/PC1‐CTF complex formation using co‐IP and PC2‐HA as a “bait” protein. PC2 and PC1‐CTF were detected using western blot in co‐IP preparations (*right panels*) and in corresponding cell lysates (*left panels*). PC2/PC1‐CTF complexes were isolated using an anti‐HA antibody conjugated to magnetic beads, which recognized HA‐tagged PC2. Original uncropped images of the same western blots are shown in Fig. S4.

Our functional results suggested that PC2 without or with PC1‐CTF co‐expression stayed mainly in the closed state at the oocyte surface. Structural information indicates that in the closed state the ion permeation pathway of the PC2 homomeric channel is occluded by two lower gate residues L677 and N681 [14, 15, 16, 17] (Fig. 1A). Similarly, in the heterotetrameric PC2/PC1 complex the putative lower gate is occluded by the same PC2 residues and additionally by three positively charged PC1 residues (R4100, R4107, H4111) [59] (Fig. 1B). It is now well‐established that alanine substitutions of L677 and N681 in PC2 produce a strong GOF effect on PC2‐mediated currents probably by removing the lower gate constriction [38, 40]. In this study, we could reproduce this GOF effect. Indeed, in oocytes expressing the GOF PC2 construct (PC2 L677A N681A, further referred to as PC2 AA) large Na^+^ inward currents were observed under divalent cation‐free conditions (Fig. 1C,F). Replacing Na^+^ with NMDG^+^ in the bath solution completely blocked these inward currents, which demonstrated that the inward current component activated by divalent cation removal was carried by Na^+^. Similar to the inhibitory effect of PC1‐CTF on PC2 currents (Fig. S1), the PC2 AA‐mediated currents were significantly reduced by co‐expressing PC1‐CTF (PC2 AA + PC1‐CTF; Fig. 1D,F). This effect was not due to reduced cell surface expression of PC2 AA in the presence of PC1‐CTF (Fig. 1H; Fig. S3). Instead, it probably resulted from the formation of PC2 AA/PC1‐CTF heteromeric complexes as suggested by our co‐immunoprecipitation experiments (Fig. 1I; Figs S4, S5). In accordance with the structural data, three positively charged residues of PC1 (R4100, R4107, and H4111) may partially occlude the putative lower gate of the heteromeric channel, thereby hindering the ion passage through the pore (Fig. 1D). Importantly, alanine substitutions of these PC1 residues (R4100A, R4107A, and H4111A) produced a novel GOF PC1‐CTF construct (further referred to as PC1‐CTF AAA), which formed complexes with PC2 AA (Fig. 1I) and dramatically increased whole‐cell Na^+^ inward currents (PC2 AA + PC1‐CTF AAA; Fig. 1E,F). Neither PC1‐CTF nor PC1‐CTF AAA expressed alone elicited measurable currents, despite their trafficking to the plasma membrane (Fig. S6). This is consistent with the interpretation that PC1‐CTF is unable to form functional homomeric ion channels, but can associate with PC2 to produce heteromeric ion channels. In additional experiments, we demonstrated that gradual substitution of PC1‐CTF by PC1‐CTF AAA in co‐expression experiments with PC2 AA gradually increased the magnitude of Na^+^ inward currents (Fig. S7A). This further confirmed the GOF effect of the alanine substitutions in PC1‐CTF.

We also noticed that the relative inhibitory effect of extracellular divalent cations on Na^+^ inward currents was significantly reduced in PC2 AA+PC1‐CTF and PC2 AA+PC1‐CTF AAA expressing oocytes compared to oocytes expressing PC2 AA alone (Fig. 1G). PC1‐CTF or PC1‐CTF AAA co‐expression reduced divalent cation sensitivity to a similar extent. This indicates that the effect is independent of the GOF alanine mutations in PC1‐CTF (Fig. S7B). Divalent cations are believed to block the ion permeation pathway of PC2 at least in part due to binding to the negatively charged D643 residues within the channel's selectivity filter [16, 38]. Thus, in PC2/PC1‐CTF heteromeric channels, the coordination of Ca^2+^ and Mg^2+^ by D643 is probably disturbed due to the incorporation of PC1‐CTF into the pore.

**Fig. 2:**
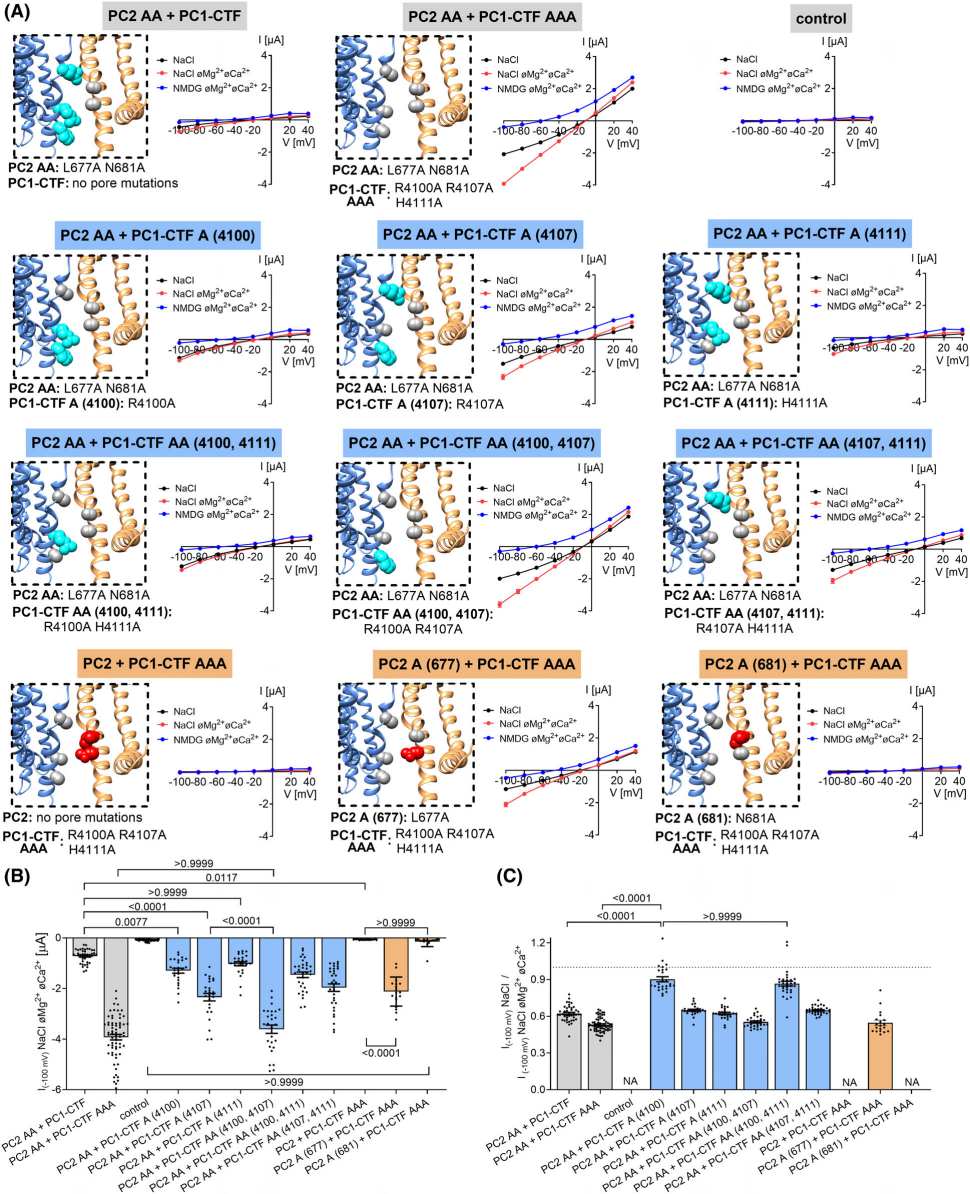
Alanine substitutions of the R4107 residue in PC1‐CTF and L677 residue in PC2 are the major contributors to the GOF effect. (A), *Left panels* Localization of alanine substitutions in gray within the pore of heteromeric PC2/PC1 ion channels introduced to create PC2 and PC1 constructs as indicated. *Right panels* Average I/V‐plots (mean ± SEM) obtained using the same experimental approach as shown in Fig. 1 from oocytes co‐expressing the corresponding PC2 (2.5 ng) and PC1‐CTF (5 ng) constructs in three different bath solutions (PC2 AA + PC1‐CTF: *n* = 44, *N* = 5; PC2 AA + PC1‐CTF AAA: *n* = 66, *N* = 7; control: *n* = 60, *N* = 7; PC2 AA + PC1‐CTF A (4100): *n* = 26, *N* = 3; PC2 AA + PC1‐CTF A (4107): *n* = 26, *N* = 3; PC2 AA + PC1‐CTF A (4111): *n* = 26, *N* = 3; PC2 AA + PC1‐CTF AA (4100, 4111): *n* = 32, *N* = 4; PC2 AA + PC1‐CTF AA (4100, 4107): *n* = 28, *N* = 4; PC2 AA + PC1‐CTF AA (4107, 4111): *n* = 32, *N* = 4; PC2 + PC1‐CTF AAA: *n* = 17, *N* = 2; PC2 A (677) + PC1‐CTF AAA: *n* = 19, *N* = 2; PC2 A (681) + PC1‐CTF AAA: *n* = 19, *N* = 2). *n* indicates the number of individual oocytes analyzed per experimental group and *N* indicates the number of different batches of oocytes. (B) Maximal currents in oocytes expressing different PC2/PC1‐CTF heteromers. The maximal inward currents measured in NaCl bath solution without divalent cations (NaCl øMg^2+^øCa^2+^) at −100 mV are shown. Data are from the same experiments as in (A). The *P*‐values were calculated by the one‐way ANOVA with Bonferroni's *post hoc* test. (C), Summary of the relative inhibitory effects of Ca^2+^ and Mg^2+^ on PC2/PC1‐CTF‐mediated sodium inward currents calculated as described in Fig. 1G. The *P*‐values were calculated by the one‐way ANOVA with Bonferroni's *post hoc* test. NA, not applicable.

In further experiments, we systematically investigated individual contributions of the R4100A, R4107A, and H4111A substitutions in PC1‐CTF to the GOF effect (Fig. 2). Taken together, these results indicated that the R4107A substitution in PC1‐CTF was mainly responsible for the GOF effect, followed by the R4100A mutation. In contrast, the contribution of the H4111 residue to the pore occlusion appeared to be minor. Consistent with the results described above (Fig. 1G; Fig. S7B), the inhibitory effect of divalent cations was reduced in all groups of oocytes co‐expressing PC2 AA with different PC1‐CTF mutants (Fig. 2C), compared to oocytes expressing PC2 AA alone (Fig. 1G). This indicated preferential formation of heteromeric PC2/PC1‐CTF ion channels in these oocytes. Furthermore, we demonstrated that co‐expression of PC1‐CTF AAA with WT PC2 did not result in measurable inward currents (Fig. 2A,B). It is therefore likely that both polycystins – PC1 and PC2 – need to change their conformation from closed to open to produce fully active PC2/PC1 heteromeric channels. Finally, we demonstrated that the L677A mutation was more important for the GOF effect than the N681A mutation (Fig. 2A,B).

In addition, we confirmed the dominant‐negative effect of PC1 on another GOF PC2 construct (F604P) described previously [40]. Indeed, the ion channel activity of PC2 F604P was completely blocked by either PC1‐CTF AAA or PC1‐CTF co‐expression, suggesting formation of nonfunctional heteromeric PC2 F604P/PC1‐CTF ion channels (Fig. S8). The F604P mutation was shown to activate a specific gating mechanism of homomeric PC2, namely a π‐α helical switch within the S6 transmembrane domain, which led to the opening of the channel's lower gate [39]. This gating mechanism does not seem to function in PC2/PC1 heteromeric channels, likely because the incorporation of PC1 into the pore region interferes with the F604P‐triggered channel opening. Thus, it is tempting to speculate that homomeric PC2 and heteromeric PC2/PC1 ion channels have distinct gating mechanisms. Moreover, the functional interaction of PC1 and PC2 may be influenced by the expression system used. Indeed, in contrast to our findings in the oocyte expression system, a robust ion channel activity has recently been described in HEK293 cells cotransfected with PC1 and PC2 F604P [57, 58]. Interestingly, similar currents were elicited in HEK293 cells co‐expressing both WT PC1 and PC2 by cytosolic application of a cholesterol derivative, 7β,27‐dihydroxycholesterol (7β,27‐DHC), which was found to be enriched in cilia [58, 69]. Activation of the PC1/PC2 complex probably occurred by binding of 7β,27‐DHC to a specific pocket within PC2 [58], which can also accommodate plasma membrane phosphoinositides [17]. Consistent with this, cytosolic application of certain phosphoinositides decreased or even abolished the 7β,27‐DHC‐mediated PC1/PC2 stimulation [58]. Taken together, these findings indicate that the ion channel function of the PC1/PC2 complex depends on the membrane lipid composition. This may help explain some of the conflicting results obtained in different expression systems, but further studies are needed to investigate this issue systematically.

In summary, we functionally validated a pore‐blocking effect of positively charged PC1 residues as predicted from structural analysis. In combination with the established PC2 AA GOF construct, alanine substitutions of these residues (R4100, R4107, and H4111) in PC1‐CTF lead to a novel PC2 AA/PC1‐CTF AAA GOF construct. It has been reported that PC2 channel activity in primary cilia is strongly voltage‐dependent with increased open probability at positive membrane potentials [34, 35]. In contrast, the putative lower gate of the GOF PC2 AA homomeric and PC2 AA/PC1‐CTF AAA heteromeric channel is probably constitutively open. Therefore, it is likely that the voltage dependence of these constructs differs from that of native polycystin channels.

### Heteromeric PC2/PC1‐CTF GOF ion channels exhibited significantly altered cation selectivity and reduced Ca^2+^ permeability compared to PC2 GOF channels

**Fig. 3:**
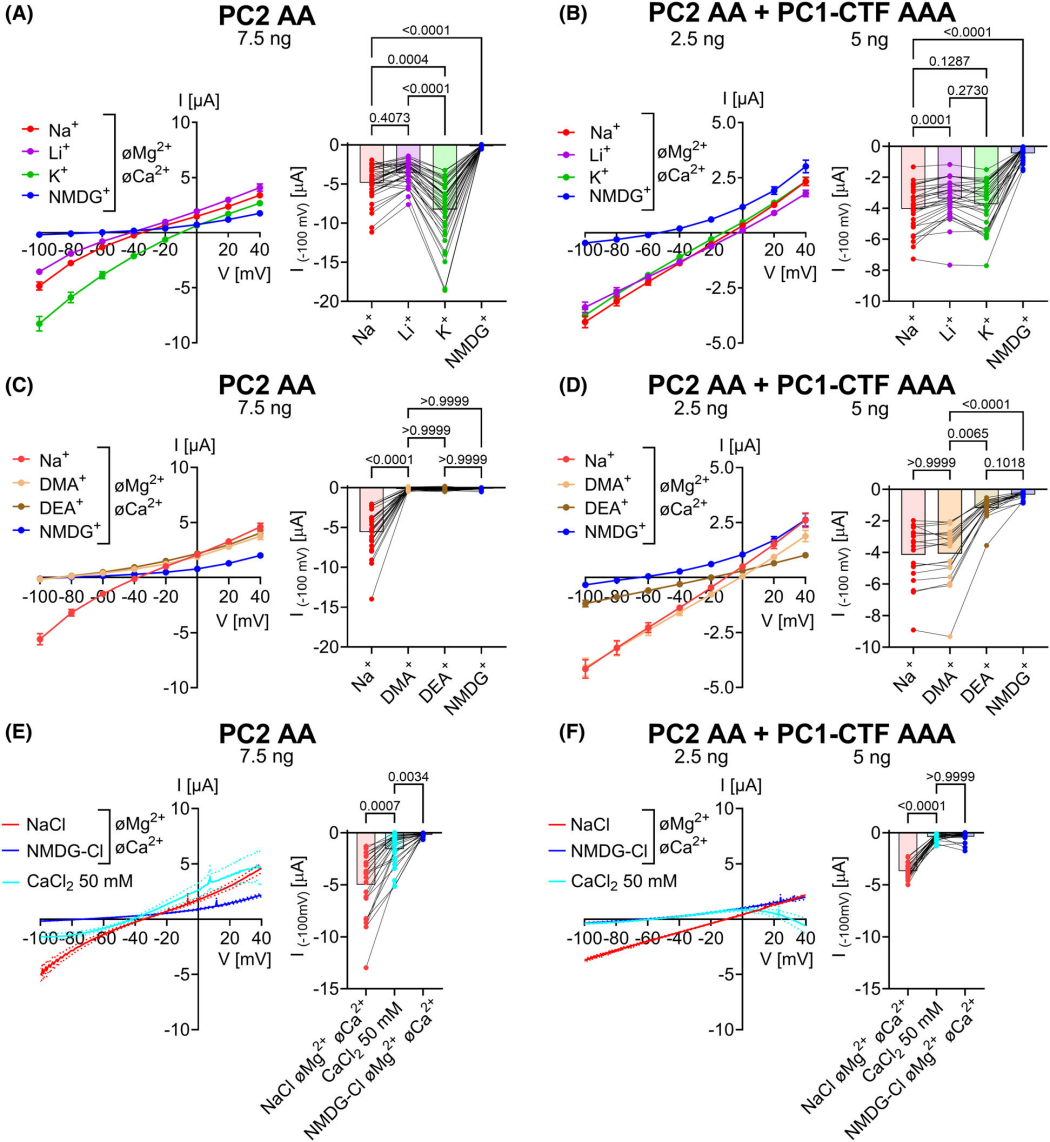
Homomeric PC2 AA and heteromeric PC2 AA/PC1‐CTF AAA ion channels demonstrate distinct cation permeability properties. (A–D), Permeability for small inorganic monovalent cations (A, B) and mid‐sized organic monovalent cations (C, D) was assessed for PC2 AA homomers (A, C) and PC2 AA/PC1‐CTF AAA heteromers (B, D) by replacing Na^+^ in the bath solution with Li^+^, K^+^, DMA^+^, DEA,^+^ or NMDG^+^ in the absence of divalent cations. The channels' permeability for Ca^2+^ (E, F) was estimated by applying 50 mm CaCl_2_ bath solution. *Left panels* Average I/V‐plots (mean ± SEM) were obtained using a similar experimental approach as shown in Fig. 1. To correct for endogenous oocyte currents, the average whole‐cell current values measured in control oocytes (Fig. S9) were subtracted from the corresponding individual whole‐cell current values measured in oocytes from the same batch expressing PC2 AA or co‐expressing PC2 AA + PC1‐CTF AAA. The I/V relationship shown in F has a negative slope at positive potentials and ultimately crosses the x‐axis at approximately +30 mV. This is an artifact introduced by subtracting corresponding current values obtained in control oocytes to correct for nonspecific leak currents (Fig. S9C). *Right panels* Summary data show maximal inward currents reached during application of hyperpolarizing pulses of −100 mV in the presence of different cations in the bath as indicated. Average values and individual data points are shown (A, *n* = 36, *N* = 3; B, *n* = 30, *N* = 3; C, *n* = 27, *N* = 3; D, *n* = 19, *N* = 3; E, *n* = 25, *N* = 3; F, *n* = 25, *N* = 3). *n* indicates the number of individual oocytes analyzed per experimental group and N indicates the number of different batches of oocytes. Lines connect data points obtained from one oocyte. The *P*‐values were calculated by the Friedman test with Dunn's *post hoc* test.

Using ion substitution experiments, we compared the cation selectivity of heteromeric PC2 AA/PC1‐CTF AAA GOF ion channels with that of PC2 AA homomers (Fig. 3). To correct for endogenous oocyte conductances, average whole‐cell current values obtained in the corresponding bath solution in control oocytes (Fig. S9) were subtracted from current values obtained in polycystin expressing oocytes from the same batch. Homomeric PC2 AA conducted K^+^ better than Na^+^, and Na^+^ slightly better than Li^+^ with no measurable conductance for NMDG^+^ (K^+^ > Na^+^ ≥ Li^+^>>>NMDG^+^; Fig. 3A) consistent with our previous report [38]. In contrast, in oocytes co‐expressing PC2 AA and PC1‐CTF AAA the measured inward currents were similar with Na^+^, Li^+^ or K^+^ as predominant cation in the bath solution. Thus, unlike PC2 AA, the heteromeric PC2 AA/PC1‐CTF AAA ion channel conducts K^+^, Na^+^ and Li^+^ equally well (K^+^ ≈ Na^+^ ≈ Li^+^>>>NMDG^+^; Fig. 3B). In agreement with this, the reversal potentials in Na^+^ or Li^+^ containing bath solutions were less negative in PC2 AA+PC1‐CTF AAA expressing oocytes than in PC2 AA expressing oocytes. This also supports the conclusion that co‐expression of PC2 AA with PC1‐CTF AAA results in ion channels which, unlike PC2 AA homomeric channels, have no preference for conducting K^+^ over Na^+^ or Li^+^. In addition, we calculated mean reversal potential shifts caused by replacing extracellular Na^+^ with K^+^, Li^+^ or NMDG^+^ to estimate cation permeability ratios (Table 1A). These estimates were in good agreement with the qualitative conclusions reached from the inward current data. It should be noted that the reversal potentials in Na^+^, Li^+^ and K^+^ containing solutions were slightly negative. This may be due to a small endogenous Cl^−^ conductance. However, switching to the NMDG^+^ bath solution nearly abolished inward currents and caused a substantial (~40 mV) negative shift of the reversal potential. This confirmed that under our experimental conditions the PC2 AA/PC1‐CTF AAA‐mediated monovalent cation conductance predominated over nonspecific endogenous Cl^−^ conductances.

**Table 1:** Effect of replacing Na^+^ in the bath solution by other small inorganic cations (A), mid‐sized organic cations (B) or a large organic cation NMDG^+^ (A and B) on average reversal potentials in *Xenopus laevis* oocytes expressing PC2 AA alone or co‐expressing PC2 AA with PC1‐CTF AAA or PC1‐CTF. Reversal potentials (in mV) observed in the presence of Na^+^ (E_rev,Na_^+^), Li^+^ (E_rev,Li_^+^), K^+^ (E_rev,K_^+^), DMA^+^ (E_rev,DMA_^+^), DEA^+^ (E_rev,DEA_^+^) or NMDG^+^ (E_rev,NMDG_^+^) were estimated from the averaged I/V‐curves shown in Fig. 3. E_rev,Na_^+^ was subtracted from E_rev,X_^+^ measured in the bath solution containing cation X^+^ (Li^+^, K^+^, DMA^+^, DEA^+^ or NMDG^+^) instead of Na^+^ to calculate the corresponding reversal potential shift (ΔE_rev,X_^+^_−Na_^+^). The reversal potential shifts were used to estimate relative permeability ratios (P_X_/P_Na_; see Eq. 1).

	PC2 AA	PC2 AA + PC1‐CTF AAA	PC2 AA + PC1‐CTF
(A) Relative permeability ratios for Na^+^, Li^+^, K^+^			
E_rev,Na_^+^ [mV]	−35	−8	−13
E_rev,Li_^+^ [mV]	−42	−3	−8
E_rev,K_^+^ [mV]	−10	−13	−15
E_rev,NMDG_^+^ [mV]	−65	−52	−58
ΔE_rev,Li_^+^_−Na_^+^ [mV]	−7	5	5
ΔE_rev,K_^+^_−Na_^+^ [mV]	25	−5	−2
ΔE_rev,NMDG_^+^_−Na_^+^ [mV]	−30	−44	−45
P_Na_ : P_Li_ : P_K_ : P_NMDG_	1.0 : 0.8 : 2.7 : 0.3	1.0 : 1.2 : 0.8 : 0.2	1.0 : 1.2 : 0.9 : 0.2
(B) Relative permeability ratios for DMA^+^, DEA^+^ and NMDG^+^			
E_rev,Na_^+^ [mV]	−38	−10	−15
E_rev,DMA_^+^ [mV]	−87	−1	−18
E_rev,DEA_^+^ [mV]	−89	−20	−46
E_rev,NMDG_^+^ [mV]	−84	−65	−71
ΔE_rev,DMA_^+^_−Na_^+^ [mV]	−49	9	−3
ΔE_rev,DEA_^+^_−Na_^+^ [mV]	−51	−10	−31
ΔE_rev,NMDG_^+^_−Na_^+^ [mV]	−46	−55	−56
P_Na_ : P_DMA_ : P_DEA_ : P_NMDG_	1.0 : 0.1 : 0.1 : 0.2	1.0 : 1.4 : 0.7 : 0.1	1.0 : 0.9 : 0.3 : 0.1

Furthermore, we investigated the permeability of homomeric PC2 AA *versus* heteromeric PC2 AA/PC1‐CTF AAA ion channels for mid‐sized organic monovalent cations (DMA^+^, DEA^+^). Homomeric PC2 GOF channels were almost impermeable to these organic cations (Na^+^>>>DMA^+^ ≈ DEA^+^ ≈ NMDG^+^; Fig. 3C; Table 1B). In contrast, heteromeric PC2/PC1‐CTF GOF channels conducted DMA^+^ and Na^+^ equally well and DEA^+^ significantly better than NMDG^+^ (Na^+^ ≈ DMA^+^ > DEA^+^ > NMDG^+^; Fig. 3D; Table 1B). Similar cation permeability properties were observed in oocytes co‐expressing PC2 AA and PC1‐CTF without alanine substitutions (PC2 AA+PC1‐CTF; Fig. S10; Table 1A,B), consistent with previous findings [40]. In our experiments, the amount of injected cRNA was increased by 3‐fold for both polycystins (PC2AA and PC1‐CTF) to achieve higher currents, thereby improving the resolution of the current measurements. From this electrophysiological analysis, we concluded that heteromerization of PC2 AA with PC1‐CTF AAA or PC1‐CTF resulted in the formation of ion channels with significantly altered ion channel properties compared to PC2 homomers. Importantly, this effect was not due to GOF alanine substitutions in the putative lower gate of PC1. Instead, it can be attributed to structural changes of the channel's selectivity filter induced by PC1.

Finally, we indirectly assessed Ca^2+^ permeability of PC2 AA and PC2 AA/PC1‐CTF AAA by applying 50 mm CaCl_2_ bath solution according to a previously established protocol [37, 38]. In this setting, the large inward currents observed at negative holding potentials are not carried by Ca^2+^ influx *per se* but result mainly from the stimulation of endogenous Ca^2+^‐activated Cl^−^ channels mediating Cl^−^ efflux. Indeed, as demonstrated previously [38], these inward currents could be largely abolished by pre‐injecting oocytes with 50 mm K‐EGTA, which effectively chelated Ca^2+^ entering the cell from the outside and prevented Cl^−^ channel activation. Thus, with this experimental approach, stimulation of Ca^2+^‐activated Cl^−^ channels can be used as a surrogate parameter to assess polycystin‐mediated Ca^2+^ influx too small to be resolved in our whole‐cell current recordings.

Consistent with our previous observation [38], in the presence of 50 mm CaCl_2_, we observed substantial inward currents in PC2 AA expressing oocytes (Fig. 3E). On average, currents measured in high Ca^2+^ solution at −100 mV were significantly larger than those measured in NMDG^+^‐containing solution. In contrast, in oocytes co‐expressing PC2 AA and PC1‐CTF AAA the inward currents measured in high Ca^2+^ bath solution did not significantly differ from those measured in NMDG^+^‐containing bath solution (Fig. 3F). Taken together, these findings indicate that PC2 AA is permeable for Ca^2+^, whereas the Ca^2+^ permeability of PC2 AA/PC1‐CTF AAA heteromers is abolished or below the detection limit of our assay. We also did not detect any permeability for Mg^2+^ or Ba^2+^ in oocytes co‐expressing PC2 AA and PC1‐CTF AAA (Fig. S11).

Next, we slightly modified our experimental protocol to enhance Ca^2+^ entry into oocytes in the presence of 50 mm CaCl_2_ in the bath solution. A holding potential of 0 mV was used instead of −60 mV to reduce the known voltage‐dependent blocking effect of Ca^2+^ on its passage through the channel's pore [36, 40]. Indeed, this modification resulted in a significantly stronger Ca^2+^‐mediated current response in PC2 AA expressing oocytes (Fig. S12A). However, under the same conditions, only a marginal Ca^2+^ permeability could be revealed in PC2 AA + PC1‐CTF AAA expressing oocytes (Fig. S12B). Interestingly, another study comparing the Ca^2+^ permeability of PC2 AA with that of PC2 AA/PC1 heteromeric ion channels came to the opposite conclusion [40]. One possible explanation for these conflicting results could be species‐specific differences. Indeed, in the previous study, mouse PC1 was co‐expressed with human PC2, whereas our study examined the human orthologs of both proteins. Mouse PC1 and human PC1 share a high degree of similarity in their primary sequence within the CTF domain (approximately 87%). Despite this, we cannot rule out the possibility that even subtle differences in the pore configuration of the heteromeric PC2/PC1 channel could significantly affect its selectivity properties. In any case, results obtained in both studies indicate that the absolute Ca^2+^ permeability of homomeric PC2 and heteromeric PC2/PC1 channels is relatively small, making precise quantification challenging. Moreover, in both studies, Ca^2+^ permeability was assessed indirectly by measuring Ca^2+^‐mediated activation of endogenous Cl^−^ channels as a readout. The relationship between Ca^2+^ influx and the elicited Cl^−^ current is likely nonlinear and variable due to differences in Cl^−^ channel expression and the rate of intracellular Ca^2+^ sequestration by Ca^2+^ pumps. Therefore, quantitative estimates of Ca^2+^ conductance of PC2 and PC2/PC1 constructs obtained using this indirect approach have to be interpreted with caution.

Taken together, our findings suggest that PC2 homomers and PC2/PC1 heteromers are two distinct types of ion channels, probably with different (patho‐)physiological roles in different tissues and subcellular localizations. This seems plausible because PC2 and PC1 demonstrate profound differences regarding their spatiotemporal expression pattern in kidney and other organs [21, 70, 71, 72, 73].

### PC2 preferentially forms heteromeric complexes with PC1‐CTF

**Fig. 4:**
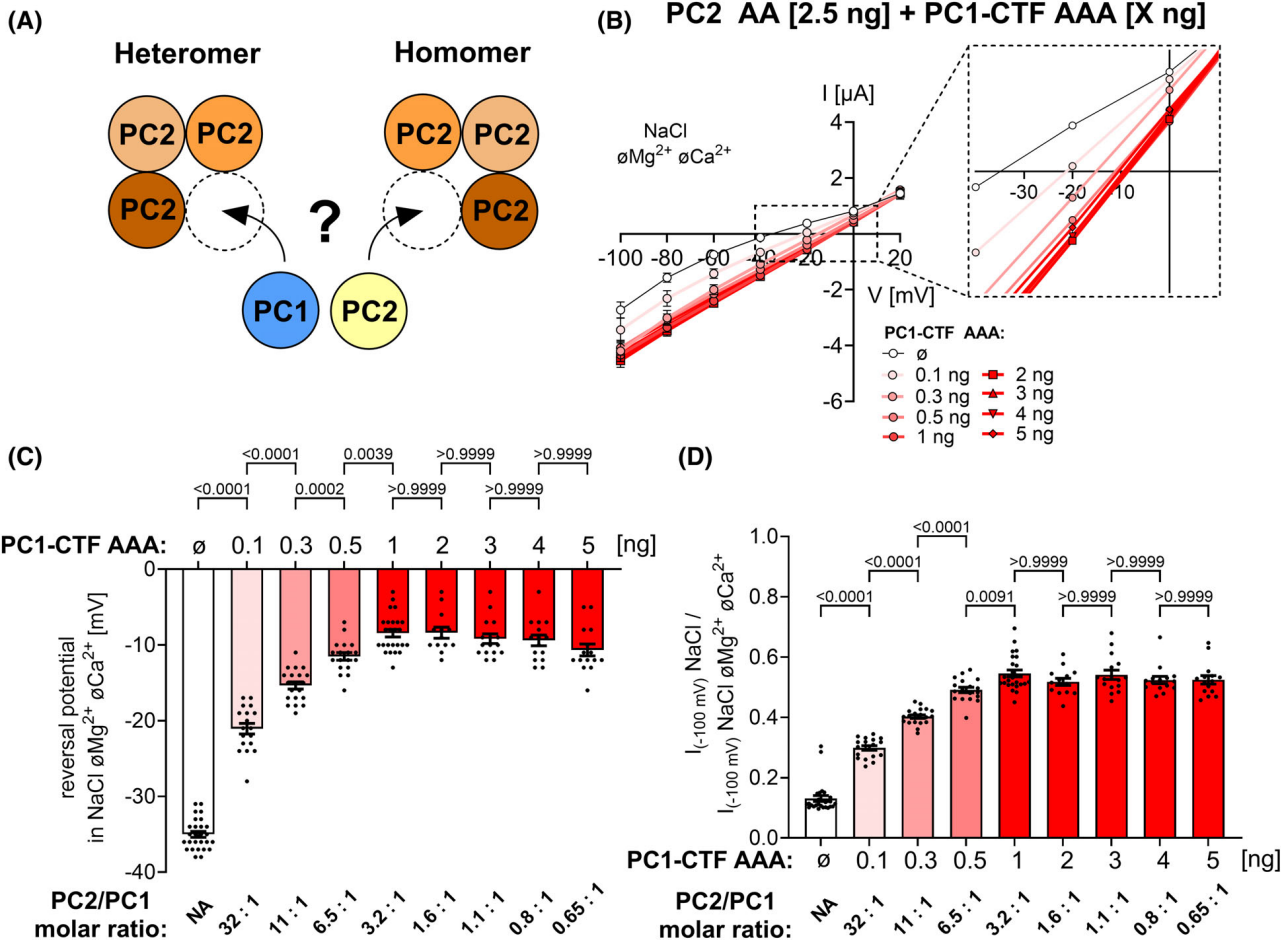
PC2 preferentially forms heterotetrameric complexes with PC1‐CTF probably in a 3 : 1 stoichiometry. (A) Schematic diagram illustrating two possibilities of PC1 and PC2 oligomerization. (B) Average I/V‐plots (mean ± SEM) obtained in NaCl bath solution without divalent cations (NaCl øMg^2+^øCa^2+^) from oocytes co‐injected with a constant amount of PC2 AA (2.5 ng) and a variable amount of PC1‐CTF AAA as indicated. The inset showing a portion of the same I/V‐plots on an expanded scale demonstrates a progressive shift of the reversal potential with increasing amount of PC1‐CTF AAA. This effect saturates at about 1 ng of injected PC1‐CTF AAA. Complete set of I/V‐plots obtained from these oocytes are shown in Fig. S13. (C) Summary of the reversal potentials from the same experiments as shown in (B). (D) Summary of the relative inhibitory effects of Ca^2+^ and Mg^2+^ on PC2AA‐ and PC2 AA/PC1‐CTF AAA‐mediated sodium inward currents calculated as described in Fig. 1G. Mean ± SEM and values from individual recordings are shown (13 ≤ *n* ≤ 27, *N* = 2–3). *n* indicates the number of individual oocytes analyzed per experimental group and *N* indicates the number of different batches of oocytes. The *P*‐values were calculated by the one‐way ANOVA with Bonferroni's *post hoc* test.

When PC1 and PC2 are co‐expressed, formation of heteromeric PC2/PC1 and homomeric PC2 complexes may occur (Fig. 4A). To determine whether heteromers or homomers are preferentially assembled in the oocyte expression system, we performed “PC1 titration” experiments by co‐expressing a fixed amount of PC2 AA with increasing amounts of PC1‐CTF AAA. Using the same experimental protocol as shown in Fig. 1, we estimated the average reversal potential in NaCl divalent cation free bath solution in each group of oocytes (Fig. 4B,C; Fig. S13). Increasing the PC1‐CTF amount resulted in a progressive shift of the reversal potential from approximately −35 mV to −10 mV. This can be explained by an increased proportion of heteromeric PC2 AA/PC1‐CTF AAA channels at the cell surface because the heteromeric channels have a reduced potassium to sodium permeability ratio compared to homomeric PC2 AA channels, in accordance with the results shown in Fig. 3A,B. Importantly, considerable heteromerization was evident even with the smallest amount of injected PC1‐CTF AAA cRNA (0.1 ng). Moreover, heteromerization reached saturation when 1 ng of PC1‐CTF AAA was co‐expressed with 2.5 ng of PC2 AA. This corresponds to 0.77 pmol and 2.5 pmol of injected cRNA encoding PC1‐CTF AAA and PC2 AA, respectively. Thus, assuming a similar translation efficiency of the cRNAs, we can estimate a PC2/PC1 molar ratio of 3.2 : 1, at which the apparent saturation of heteromerization was reached. This estimate is in good agreement with the 3 : 1 PC2/PC1 stoichiometry observed in the cryo‐EM structure [59] and with previous biochemical and biophysical results [54]. Furthermore, data from the same experiments were re‐analyzed to calculate the relative inhibitory effect of Ca^2+^ on sodium inward currents in each group of oocytes (Fig. 4D). As shown in Fig. 1G, homomeric PC2 AA channels demonstrated significantly higher sensitivity to extracellular Ca^2+^ compared to heteromeric PC2 AA/PC1‐CTF AAA channels. Therefore, in addition to the reversal potential shift, the reduction in Ca^2+^ sensitivity can be used as a parameter to estimate the dependence of the PC2/PC1 heteromerization process on PC1 dosage. Indeed, both parameters demonstrated essentially the same dependence on PC1‐CTF AAA dosage (Fig. 4D). Taken together, these results demonstrate that PC2 preferentially forms heteromeric complexes when co‐expressed with PC1‐CTF most likely with a 3 : 1 stoichiometry.

### Pore‐forming domain of PC1 exhibits a canonical TRP‐like conformation

**Fig. 5:**
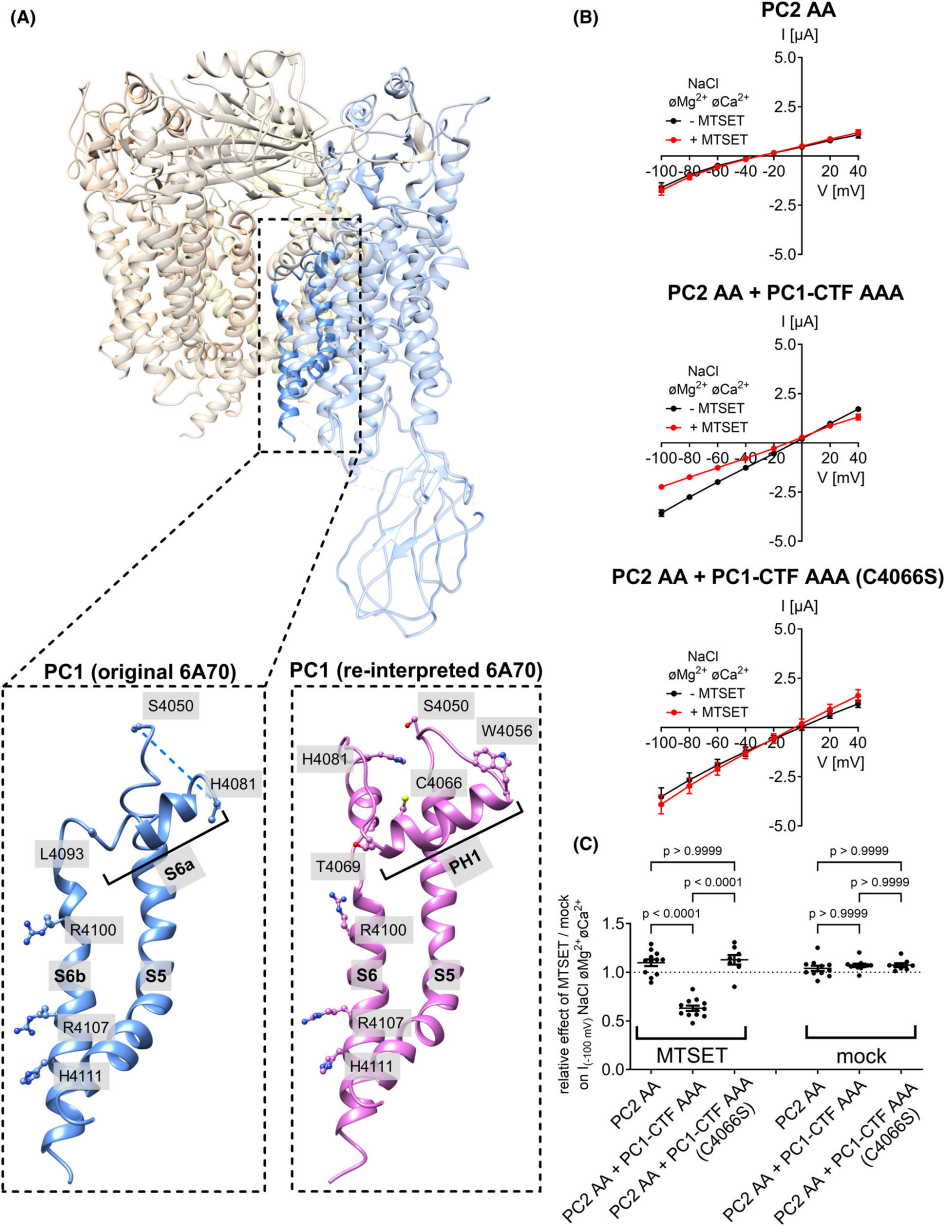
The pore domain of PC1 exhibits a canonical TRP‐like conformation. (A) Side view of human heterotetrameric PC2/PC1 complex in ribbon representation generated using the published atom coordinates from PDB entry 6A70 [59]. Individual protomers are colored similarly as in Fig. 1B. The inset shows the pore domain of PC1 on an expanded scale. The corresponding cryo‐EM data was re‐interpreted (Fig. 6) to generate a new model of the PC1 pore domain shown to the right of the published model. PH1, pore helix 1. (B) Average I/V‐plots (mean ± SEM) obtained in the NaCl bath solution without divalent cations (NaCl øMg^2+^øCa^2+^) from oocytes expressing PC2 AA alone, co‐expressing PC2 AA and PC1‐CTF AAA, or co‐expressing PC2 AA and PC1‐CTF AAA with additional C4066S mutation before (−MTSET) or after 3‐min incubation in NaCl bath solution supplemented with 1 mm MTSET (+MTSET). Complete set of I/V‐plots obtained from these oocytes are shown in Fig. S14. The oocyte was unclamped during the incubation time. Before the second current measurement, MTSET was washed out with NaCl bath solution. Impaling microelectrodes were not removed from the oocyte until the end of the experiment. (C), Summary data (mean ± SEM and individual data points) from experiments shown in B and from similar experiments performed with mock‐treated control oocytes shown in Fig. S14 are presented. Relative effects of MTSET/mock incubation on the maximal inward currents reached at −100 mV in divalent free NaCl (NaCl øMg^2+^øCa^2+^) bath solution were calculated. In each individual recording, the current measured after MTSET or mock incubation was normalized to the current measured before the treatment (8 ≤ *n* ≤ 12, *N* = 2–3). *n* indicates the number of individual oocytes analyzed per experimental group and *N* indicates the number of different batches of oocytes. The *P*‐values were calculated by the one‐way ANOVA with Bonferroni's *post hoc* test.

The published structure of the PC2/PC1 heterotetrameric complex suggests that the PC1 subunit has a noncanonical architecture of the pore‐forming domains [59]. Unlike TRP channels, such as PC2 homomers [15, 16], PC2L1 homomers [74, 75], and PC2L1/PC1L3 heteromers [76], it lacks the two pore loop helices (PH1 and PH2). Interestingly, the proximal portion of the pore loop (C4051‐W4080) was not resolved in the original model (Fig. 5A, *left structure*; PDB ID: 6A70). Additionally, the distal part of the pore loop, which resembled pore helix 1 (PH1), directly transitions into the S6 transmembrane domain and was thus interpreted as the extracellular portion of the S6 helix–S6a (Fig. 5A). We have re‐interpreted the original cryo‐EM volume (EMD‐6991) and generated an alternative model of the PC2/PC1 complex that differs significantly from the initial interpretation in certain aspects. Model‐versus‐data comparisons indicate that the new model aligns with the original cryo‐EM volume more accurately than the previous model does (Table S2).

**Fig. 6:**
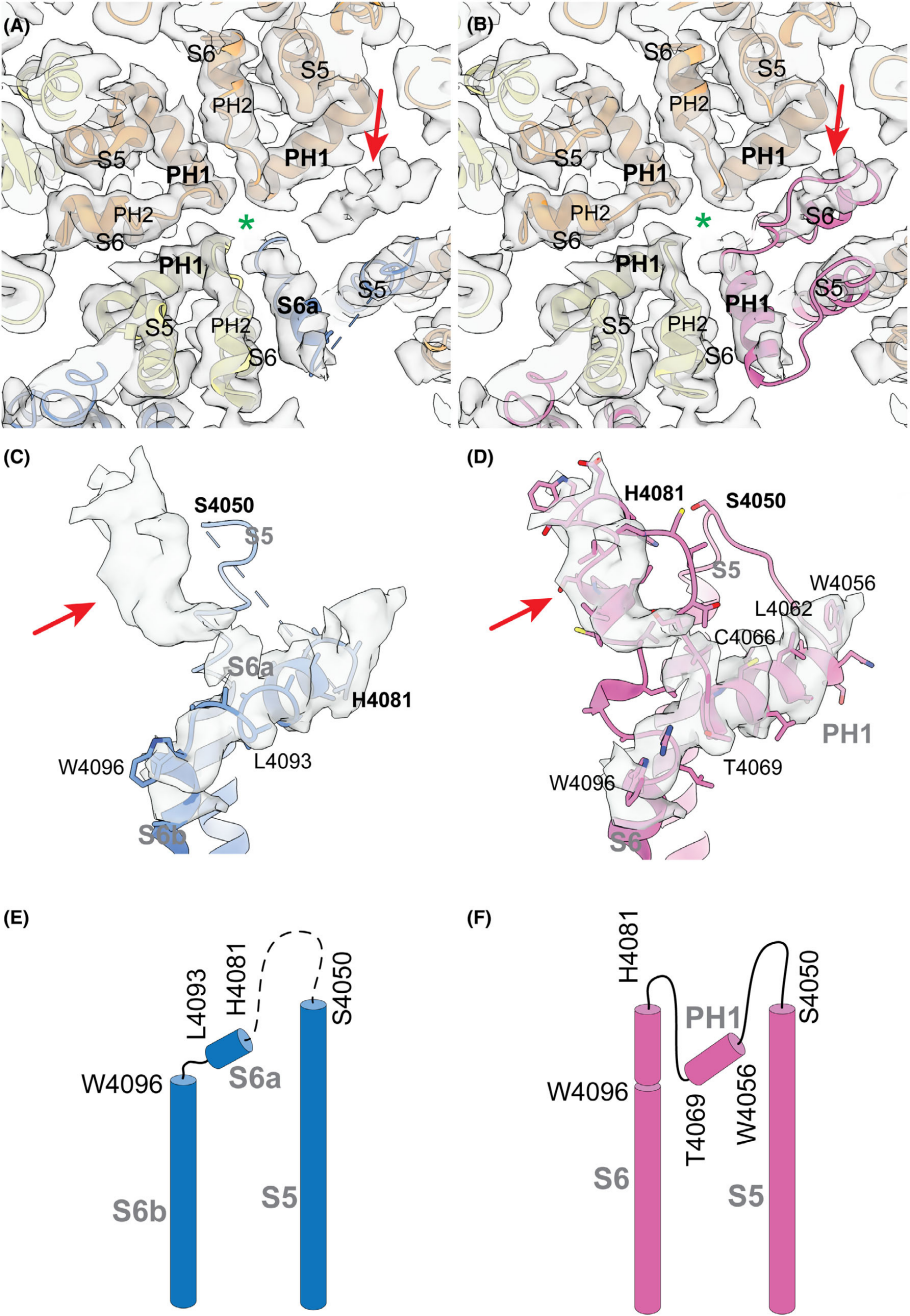
Re‐interpretation of the published cryo‐EM data. (A, B) View along the central cavity (indicated by the green color asterisk) on the selectivity filter region of the original model (A, PDB ID 6A70) or the re‐interpreted model (B) of the PC2/PC1 heterotetrameric complex. PC2 subunits are colored as in Fig. 1B, PC1 is in blue in A and in pink in B. The respective EM density (EMD‐6991) is shown as transparent volume. The red arrow indicates an unassigned, cylindrical density. (C, D) Side view on the structural elements S5‐S6a‐S6b of the original model of PC1 (C) or on the S5‐PH1‐S6 elements of the re‐interpreted model of PC1 (D). The respective PC1 EM density is shown as transparent volume. Several residues are labeled for orientation. Not modeled residues in C are indicated by a broken line. Side chains are shown in stick representation (for S6a in C side chains are not present according to the original interpretation). The red arrow points to the same unassigned density as indicated in A and B, E, F Secondary structure diagrams of the original (E) and the re‐interpreted model (F) of PC1.

We identified an unassigned cylindrical density in the pore‐forming region of the PC2/PC1 complex (Fig. 6A–D, indicated by red arrow). This discovery led us to re‐assess the EM densities of the pore‐forming domains of PC1 by using a homology model of PC1, which included the S5‐pore loop‐S6 segments and was based on the PC2 structure (26.11% sequence identity). Refining this model against the published EM map suggested a plausible configuration of PC1, wherein a canonical TRP‐like arrangement of the S5‐PH1‐S6 segments is feasible (Fig. 5A, *right structure*; Fig. 6B,D,F). We interpreted the previously unassigned cylindrical density as part of the continuous S6 helix (H4081‐V4088; Fig. 6B,D,F), indicating that the S6 helix likely remains intact and is not divided into S6a and S6b sections, as initially proposed. Additionally, the more proximal sequence (W4056‐T4069) with its medium‐sized side chains aligns better with the putative PH1 EM density than the originally suggested H4081‐L4093 sequence (Fig. 6A,C,E).

We identified a density to accommodate the C4066 near the distal end of PH1 (Fig. 6D; Fig. 5A, *right structure*). Importantly, functional experiments using a sulfhydryl reagent MTSET suggested that the C4066 residue is accessible for covalent modification consistent with the revised structural model. Indeed, in oocytes co‐expressing PC2 AA and PC1‐CTF AAA, MTSET incubation led to a significant and irreversible decrease in currents (Fig. 5B,C, Fig. S14). This effect was not observed in mock control experiments or in oocytes expressing PC2 AA alone. Notably, the inhibitory effect of MTSET was absent in oocytes co‐expressing PC2 AA and a C4066S mutant of PC1‐CTF AAA (Fig. 5B,C; Fig. S14). Application of DTT completely reversed the inhibitory effect of MTSET on PC2 AA/PC1‐CTF AAA channels (Fig. S15), likely by removing the adduct from the cysteine residue [77]. In addition, we generated a theoretical PC1/PC2 heterocomplex structure using AlphaFold 3 [78]. The AI‐generated structure was consistent with our re‐interpretation of the structural data, but differed from the originally reported model of PC1 (Fig. S16). These results further support our conclusion that the pore loop of PC1 has a canonical TRP‐like conformation.

In summary, our findings provide strong evidence that PC1‐CTF and PC2 can form heteromeric ion channels with ion channel properties different from those of PC2 homomeric channels. We validated the published structure of the PC2/PC1 complex by generating a novel GOF PC2/PC1‐CTF heteromeric ion channel (PC2 AA/PC1‐CTF AAA). This mutated channel likely possesses a constitutively open ion permeation pathway and does not require active gating to achieve an open state. Consequently, it is unsuitable for studying the effects of ADPKD‐associated mutations in PC2 and PC1 on the gating mechanism of the heteromeric channel. However, this PC2/PC1 GOF channel may serve as a valuable tool for investigating whether ADPKD‐associated mutations disrupt the oligomerization and cation conductance of heteromeric PC2/PC1 ion channel complexes. These future investigations may enhance our understanding of the role of PC2/PC1 ion channels in the pathogenesis of ADPKD and support the concept that ADPKD is a channelopathy [37, 38, 41, 79].

## Author contributions

T.S., C.K., and A.V.I. planned experiments; T.S., J.K., L.G., B.A., and A.V.I. performed experiments; T.S., M.G.M., C.Z., C.K., and A.V.I. analyzed data; T.S., M.G.M., C.Z., C.K., and A.V.I. wrote the paper.

## Peer review

The peer review history for this article is available at https://www.webofscience.com/api/gateway/wos/peer‐review/10.1002/1873‐3468.70059.

## Supporting information


**Fig. S1.** Ion channel activity of PC2 expressed alone or together with PC1‐CTF is low.
**Fig. S2.** Cell surface and intracellular expression of PC2 and PC1‐CTF.
**Fig. S3.** Cell surface and intracellular expression of PC2 AA, PC1‐CTF and PC1‐CTF AAA.
**Fig. S4.** Validation of PC2/PC1‐CTF complex formation using co‐IP and PC2‐HA as a “bait” protein.
**Fig. S5.** Validation of PC2/PC1‐CTF complex formation using co‐IP and PC1‐V5 as a “bait” protein.
**Fig. S6.** Expression of PC1‐CTF or PC1‐CTF AAA alone did not result in detectable ion channel currents.
**Fig. S7.** Replacing PC1‐CTF by PC1‐CTF AAA in co‐expression experiments with PC2 AA increased ion channel currents without changing the inhibitory effect of divalent cations.
**Fig. S8.** Ion channel function of PC2 F604P is blocked by PC1‐CTF or PC1‐CTF AAA co‐expression.
**Fig. S9.** Effect of monovalent cation substitutions and application of 50 mm CaCl_2_ bath solution on baseline currents in control oocytes.
**Fig. S10.** Permeability for monovalent cations of heteromeric PC2 AA/PC1‐CTF ion channels.
**Fig. S11.** Heteromeric PC2 AA/PC1‐CTF AAA ion channels are impermeable for divalent cations Mg^2+^ and Ba^2+^.
**Fig. S12.** Heteromeric PC2 AA/PC1‐CTF AAA ion channels are less permeable for Ca^2+^ than PC2 AA homomers even under experimental conditions facilitating Ca^2+^ entry.
**Fig. S13.** Average I/V plots from co‐expression experiments using a fixed amount of PC2 AA and increasing amounts of PC1‐CTF AAA.
**Fig. S14.** Sulfhydryl reagent MTSET inhibits PC2 AA/PC1‐CTF AAA heteromers through covalent modification of the pore loop residue C4066.
**Fig. S15.** Inhibitory effect of MTSET on ion channel function of PC2 AA/PC1‐CTF AAA heteromers can be rescued by DTT.
**Fig. S16.** AlphaFold 3‐generated model of the PC2/PC1 heterocomplex.
**Table S1.** Reagents and Tools.
**Table S2.** Comparison of the model‐versus‐data metrics for PC1/PC2 heterocomplex of the published (PDB ID: 6A70) and re‐interpreted model (this study).

## Data Availability

The data that support the findings of this study are available in Figs 1, 2, 3, 4, 5, 6, Table 1, and the supplementary material of this article.
