# Supplementary material for: Ion channel function of polycystin‐2/polycystin‐1 heteromer revealed by structure‐guided mutagenesis
Source: FEBS Lett. 2025 May 12;599(12):1649–68. doi: 10.1002/1873-3468.70059 (PMC12183636; doi:10.1002/1873-3468.70059)
Supplement: Supplementary file 1 — Fig. S1. Ion channel activity of PC2 expressed alone or together with PC1‐CTF is low. Fig. S2. Cell surface and intracellular expression of PC2 and PC1‐CTF. Fig. S3. Cell surface and intracellular expression of PC2 AA, PC1‐CTF and PC1‐CTF AAA. Fig. S4. Validation of PC2/PC1‐CTF complex formation using co‐IP and PC2‐HA as a “bait” protein. Fig. S5. Validation of PC2/PC1‐CTF complex formation using co‐IP and PC1‐V5 as a “bait” protein. Fig. S6. Expression of PC1‐CTF or PC1‐CTF AAA alone did not result in detectable ion channel currents. Fig. S7. Replacing PC1‐CTF by PC1‐CTF AAA in co‐expression experiments with PC2 AA increased ion channel currents without changing the inhibitory effect of divalent cations. Fig. S8. Ion channel function of PC2 F604P is blocked by PC1‐CTF or PC1‐CTF AAA co‐expression. Fig. S9. Effect of monovalent cation substitutions and application of 50 mm CaCl2 bath solution on baseline currents in control oocytes. Fig. S10. Permeability for monovalent cations of heteromeric PC2 AA/PC1‐CTF ion channels. Fig. S11. Heteromeric PC2 AA/PC1‐CTF AAA ion channels are impermeable for divalent cations Mg2+ and Ba2+. Fig. S12. Heteromeric PC2 AA/PC1‐CTF AAA ion channels are less permeable for Ca2+ than PC2 AA homomers even under experimental conditions facilitating Ca2+ entry. Fig. S13. Average I/V plots from co‐expression experiments using a fixed amount of PC2 AA and increasing amounts of PC1‐CTF AAA. Fig. S14. Sulfhydryl reagent MTSET inhibits PC2 AA/PC1‐CTF AAA heteromers through covalent modification of the pore loop residue C4066. Fig. S15. Inhibitory effect of MTSET on ion channel function of PC2 AA/PC1‐CTF AAA heteromers can be rescued by DTT. Fig. S16. AlphaFold 3‐generated model of the PC2/PC1 heterocomplex. Table S1. Reagents and Tools. Table S2. Comparison of the model‐versus‐data metrics for PC1/PC2 heterocomplex of the published (PDB ID: 6A70) and re‐interpreted model (this study). [file FEB2-599-1649-s001.pdf]

Supporting Information

Ion channel function of polycystin-2/polycystin-1 heteromer revealed by structure-guided mutagenesis

Tobias Staudner, Juthamas Khamseekaew, M. Gregor Madej, Linda Geiges, Bardha Azemi, Christine Ziegler, Christoph Korbmacher and Alexandr V. Ilyaskin

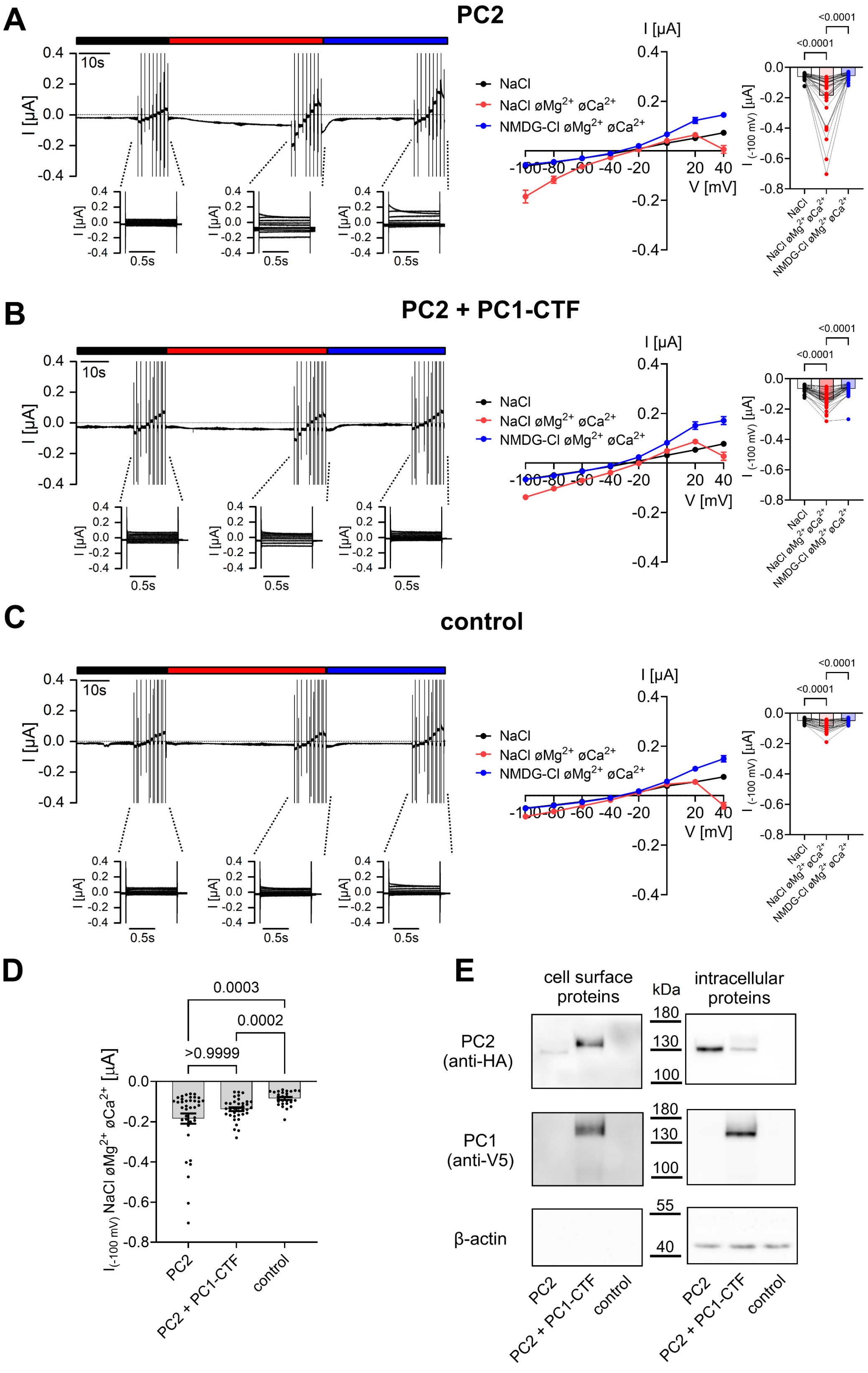
A
PC2
B
PC2 + PC1-CTF
C
control
NaCl
NaCl øMg2+ øCa2+
NMDG-Cl øMg2+ øCa2+
I [µA]
V [mV]
10s
0.5s
<0.0001
D
0.0003
0.0002
>0.9999
PC2
PC2 + PC1-CTF
control
E
cell surface proteins
intracellular proteins
kDa
PC2 (anti-HA)
PC1 (anti-V5)
β-actin
180
130
100
55
40

Fig. S1. Ion channel activity of PC2 expressed alone or together with PC1-CTF is low. ***A*-*C***, *Left panels* Representative whole-cell current trace obtained in an oocyte injected with 7.5 ng cRNA encoding human PC2 alone (***A***), or with additional co-injection of 15 ng cRNA encoding PC1-CTF (***B***), or solely injected with AS Cx38 co-injected in all oocytes to suppress endogenous Cx38 expression (***C***). Presence of standard NaCl bath solution with or without divalent cations (øMg^{2+}øCa^{2+}) or NMDG-Cl bath solution without divalent cations is indicated by black, red, and blue bars, respectively. Overlays of whole-cell current traces resulting from voltage step protocols are shown below the continuous current recordings. *Middle panels* Average I/V-plots (mean ± SEM) were constructed from similar recordings as shown in *left panels* (***A***: *n* =37, N=3; ***B***: *n* =36, N=3; ***C***: *n* =26, N=2). *n* indicates the number of individual oocytes analyzed per experimental group and N indicates the number of different batches of oocytes. *Right panels* Summary data of the same experiments as shown in (***A***-***C***). The maximal inward currents reached during the application of hyperpolarizing pulses of -100 mV in three different bath solutions are shown. Lines connect data points obtained from one oocyte. The *p*-values were calculated by the Friedman test with Dunn's post hoc test. ***D***, Maximal currents in PC2 *vs.* PC2/PC1-CTF expressing oocytes. The maximal inward currents measured in NaCl bath solution without divalent cations (NaCl øMg^{2+}øCa^{2+}) at -100 mV are shown. Data are from the same experiments as summarized in the I/V plots shown in (***A***-***C***). The *p*-values were calculated by the Kruskal-Wallis test with Dunn's post hoc test. ***E***, Western blot analysis of cell surface (*left panels*) and intracellular (*right panels*) expression of PC2 and PC1-CTF in oocytes from one batch. Original uncropped images of the same blots are shown in Fig.S2.

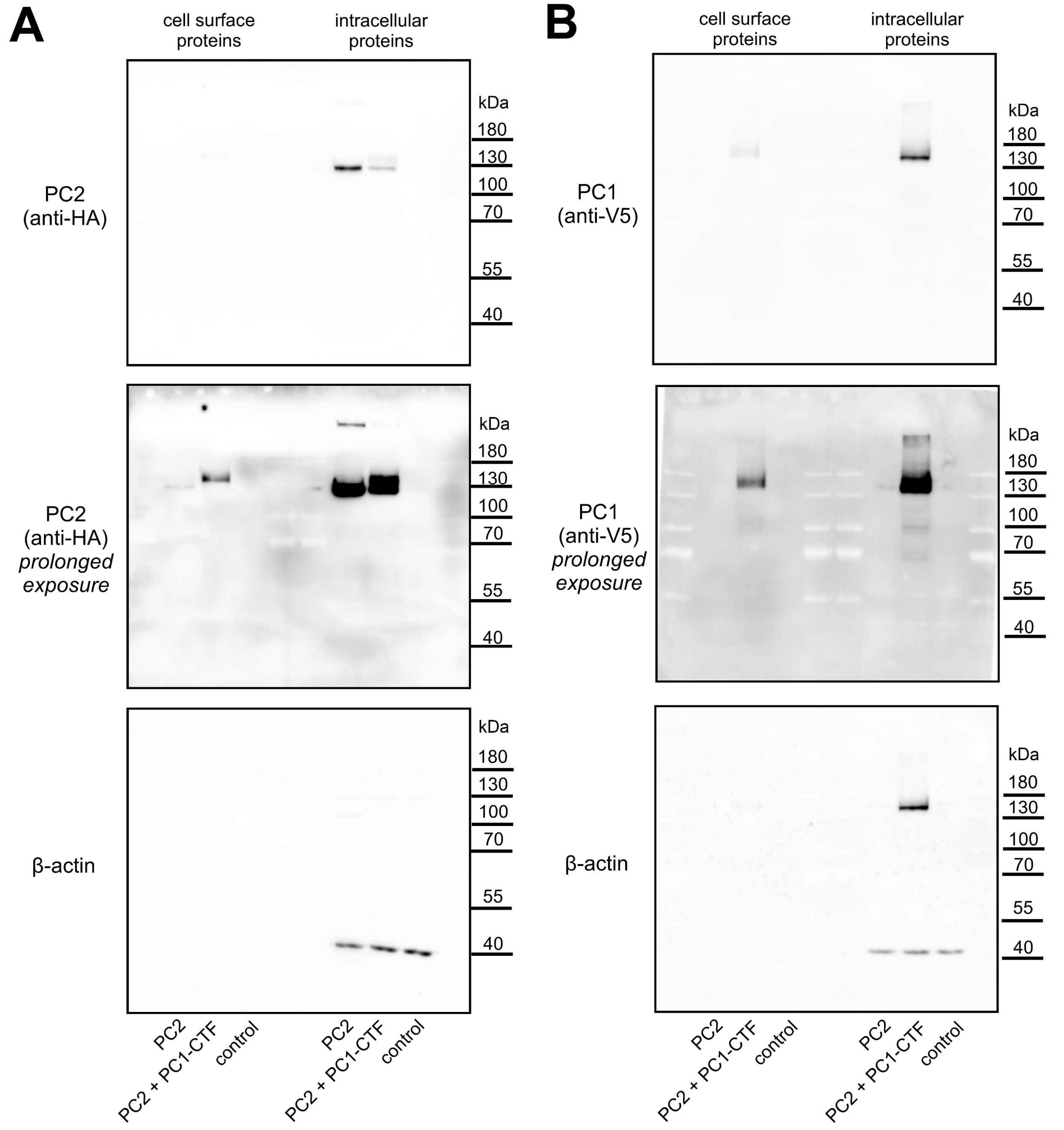

Fig. S2. Cell surface and intracellular expression of PC2 and PC1-CTF. ***A***, ***B*** Original uncropped images of the same western blots shown in Figure S1-***E***. Images showing PC2 (***A****, upper* and *middle panels*) or PC1-CTF (***B****, upper* and *middle panels*) signals were obtained using short (*upper panels*) or prolonged (*middle panels*) exposure for optimal detection of intracellular or cell surface expression, respectively. To confirm separation of cell surface proteins from intracellular proteins, the membranes were stripped and re-probed using an anti-β-actin antibody (*lower panels*).

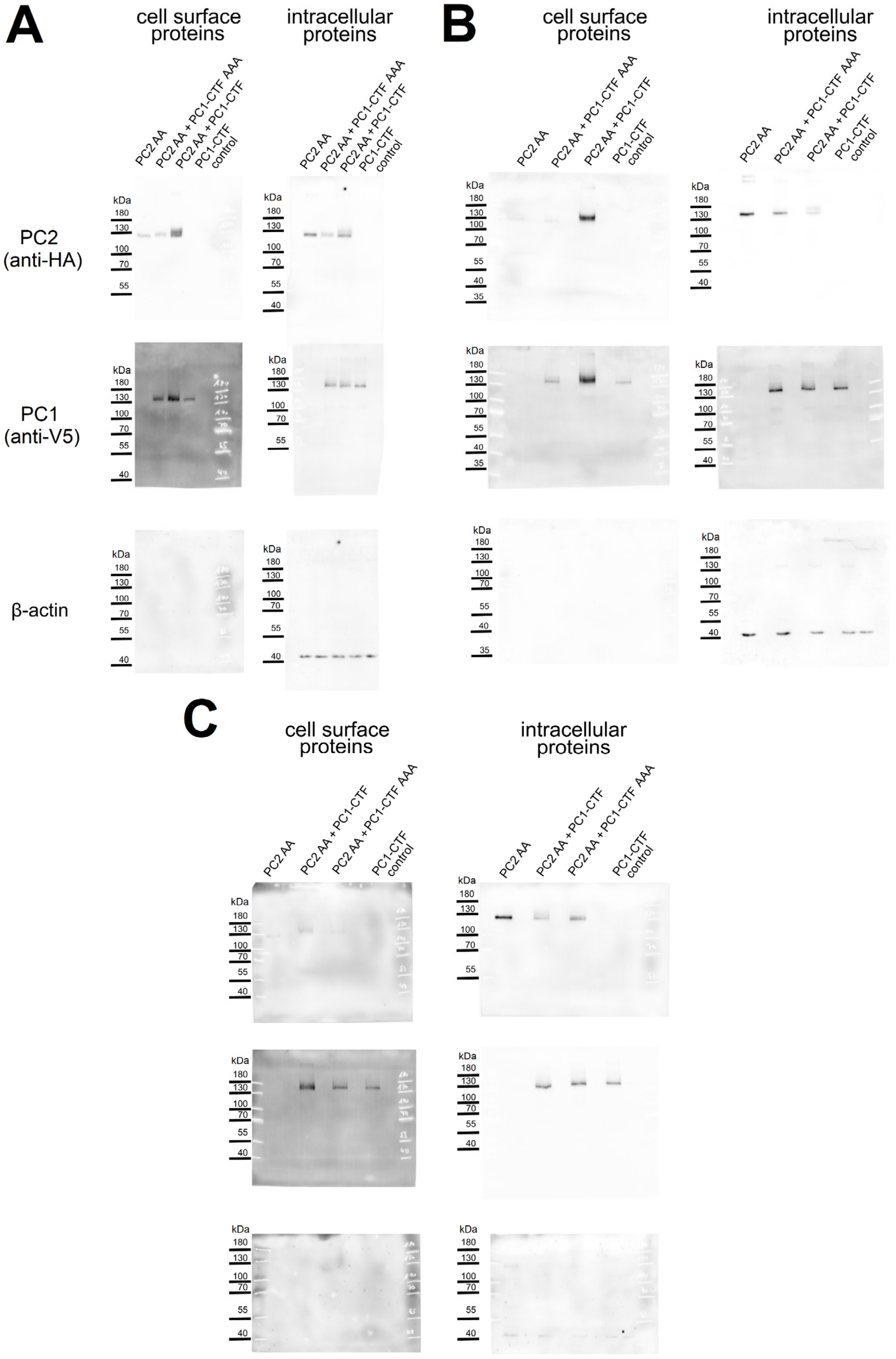

Fig. S3. Cell surface and intracellular expression of PC2 AA, PC1-CTF and PC1-CTF AAA. Original uncropped western blot images obtained in oocytes from three different batches (***A***: batch 1; ***B***: batch 2; ***C***: batch 3). The data from the batch 1 (***A***) are included in Fig. 1 as panel ***H***.

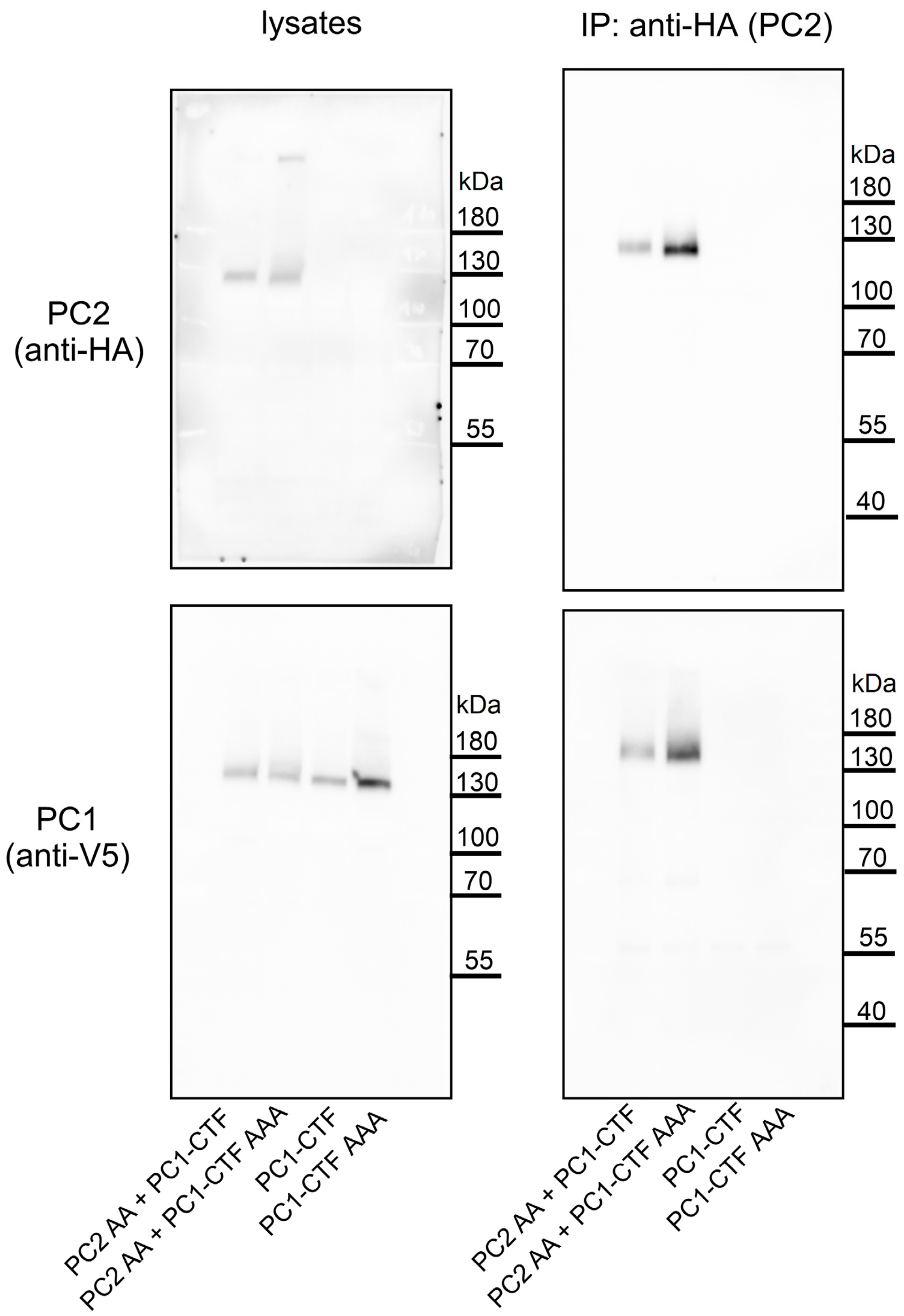

Fig. S4. Validation of PC2/PC1-CTF complex formation using co-IP and PC2-HA as a "bait" protein. Original uncropped images of the same western blots as shown in Figure 1-*I*.

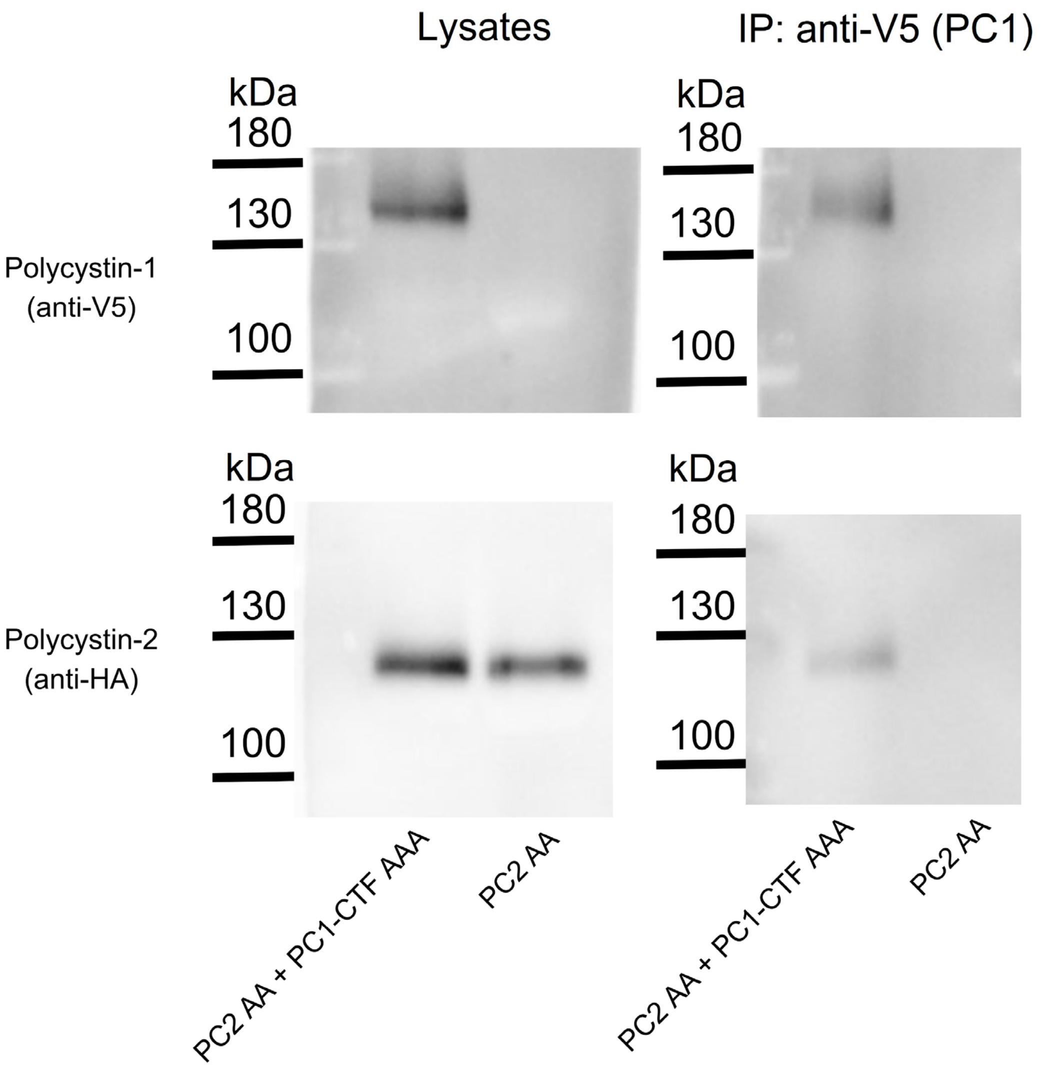

Fig. S5. Validation of PC2/PC1-CTF complex formation using co-IP and PC1-V5 as a "bait" protein. PC2 and PC1-CTF were detected in co-IP preparations (right panels) and in corresponding cell lysates (left panels). PC2/PC1-CTF complexes were isolated using an anti-V5 antibody conjugated to magnetic beads, which recognized V5-tagged PC1-CTF.

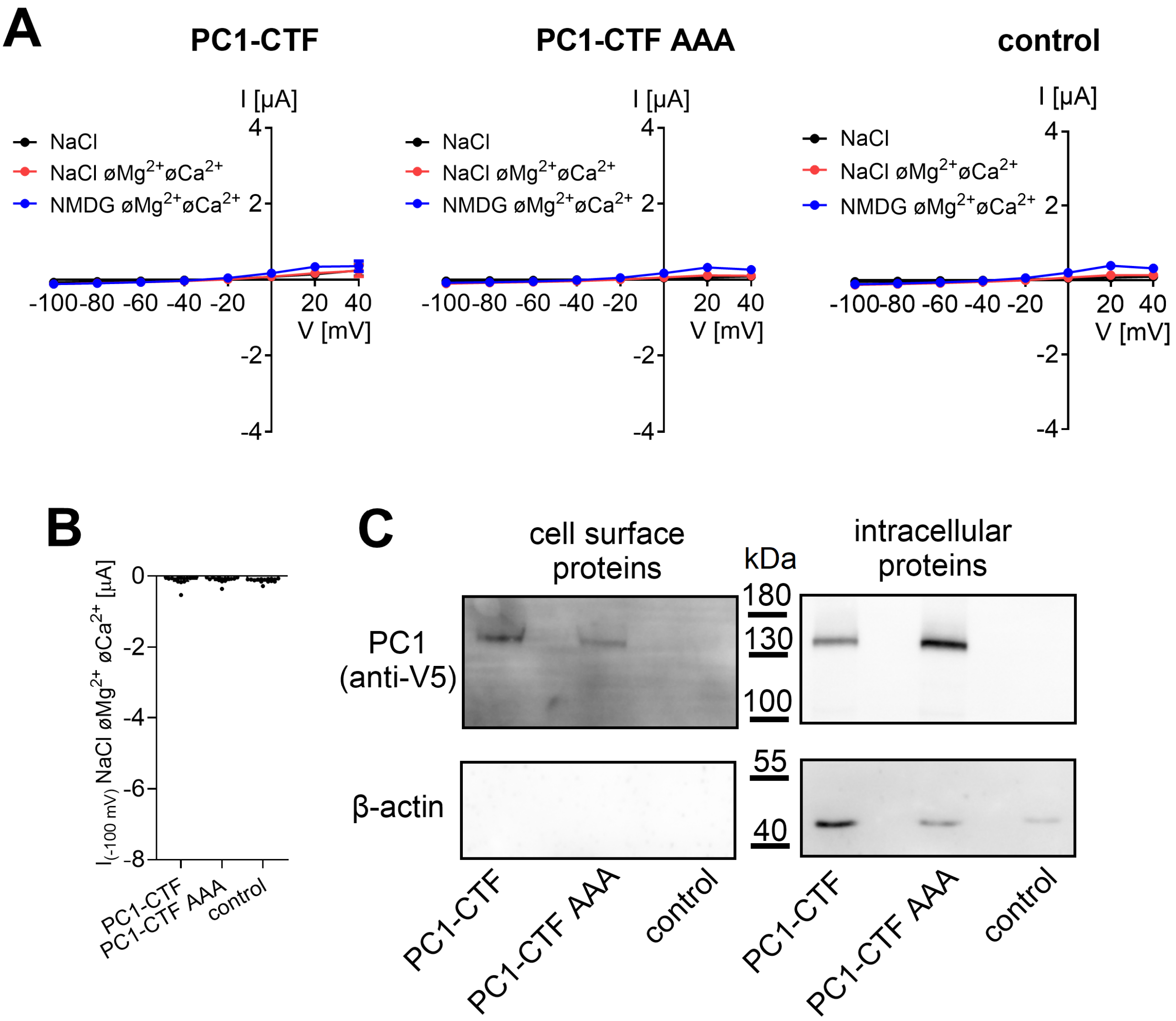

Fig. S6. Expression of PC1-CTF or PC1-CTF AAA alone did not result in detectable ion channel currents. ***A***, Average I/V-plots (mean ± SEM) obtained from oocytes injected with 15 ng of PC1-CTF or PC1-CTF AAA encoding cRNA or from control oocytes (*n* =15, N=2) using the same experimental approach as shown in Fig. 1. *n* indicates the number of individual oocytes analyzed per experimental group and N indicates the number of different batches of oocytes. ***B***, Maximal inward currents measured in NaCl bath solution without divalent cations (NaCl $øMg^{2+}øCa^{2+}$) at -100 mV. Data are from the same experiments as in (***A***). ***C***, Western blot analysis of cell surface (*left panels*) and intracellular (*right panels*) expression of PC1-CTF and PC1-CTF AAA in oocytes from one batch.

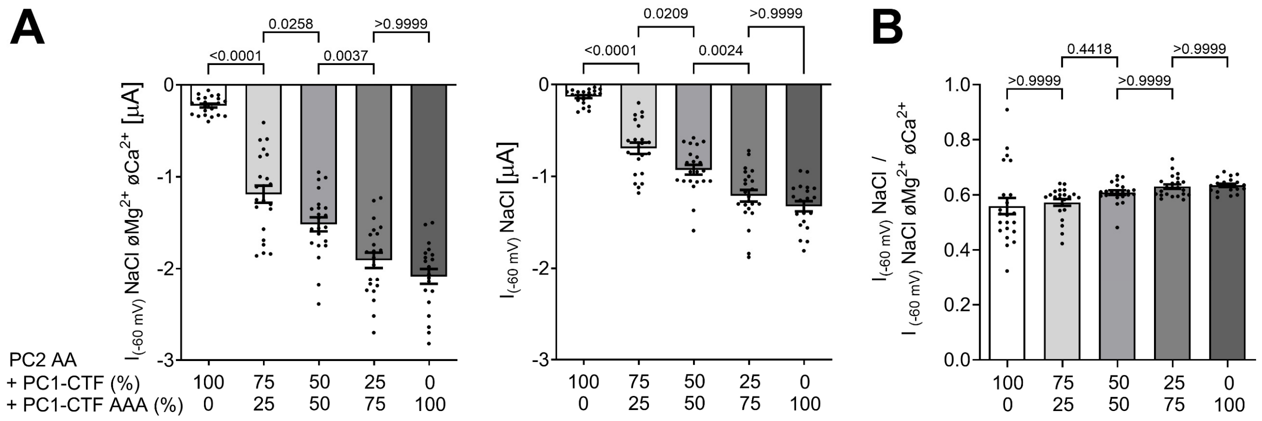

Fig. S7. Replacing PC1-CTF by PC1-CTF AAA in co-expression experiments with PC2 AA increased ion channel currents without changing the inhibitory effect of divalent cations. *A*, Inward currents were obtained in NaCl bath solution without (*left panel*) or with divalent cations (NaCl, *right panel*) from oocytes co-injected with a constant amount of PC2 AA cRNA (2.5 ng) and variable relative amounts of PC1-CTF or PC1-CTF AAA cRNAs (mean ± SEM; $20 \leq n \leq 22$, N=2). *n* indicates the number of individual oocytes analyzed per experimental group and N indicates the number of different batches of oocytes. The total cRNA amount of PC1-CTF+PC1-CTF AAA was kept constant at 5 ng. The relative amounts of PC1-CTF and PC1-CTF AAA cRNAs (in %) are indicated on the x-axis. The *p*-values were calculated by the one-way ANOVA with Bonferroni's post hoc test. ***B***, Summary of the relative inhibitory effects of Ca^{2+} and Mg^{2+} on PC2/PC1-CTF-mediated sodium inward currents. The *p*-values were calculated by the Kruskal-Wallis test with Dunn's post hoc test.

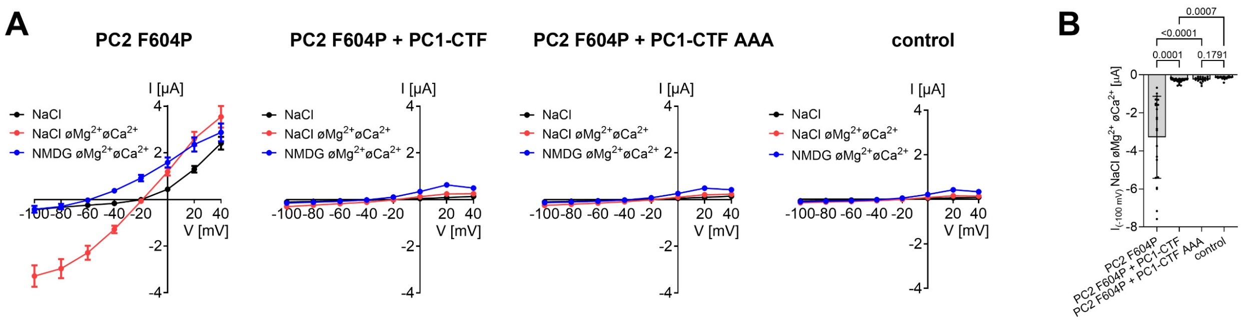

Fig. S8. Ion channel function of PC2 F604P is blocked by PC1-CTF or PC1-CTF AAA co-expression. ***A***, Average I/V-plots (mean ± SEM) obtained using the same experimental approach as shown in Fig. 1 from oocytes injected with 7.5 ng of cRNA encoding PC2 F604P only, co-injected with 15 ng of cRNA encoding PC1-CTF or PC1-CTF AAA, or from control oocytes (PC2 F604P: *n* =22, N=3; PC2 F604P + PC1-CTF: *n* =26, N=3; PC2 F604P + PC1-CTF AAA: *n* =20, N=3; control: *n* =23, N=3). *n* indicates the number of individual oocytes analyzed per experimental group and N indicates the number of different batches of oocytes. ***B***, Maximal inward currents measured in NaCl bath solution without divalent cations (NaCl øMg^{2+}øCa^{2+}) at -100 mV. Data are from the same experiments as in (***A***). The *p*-values were calculated by the Kruskal-Wallis test with Dunn's post hoc test.

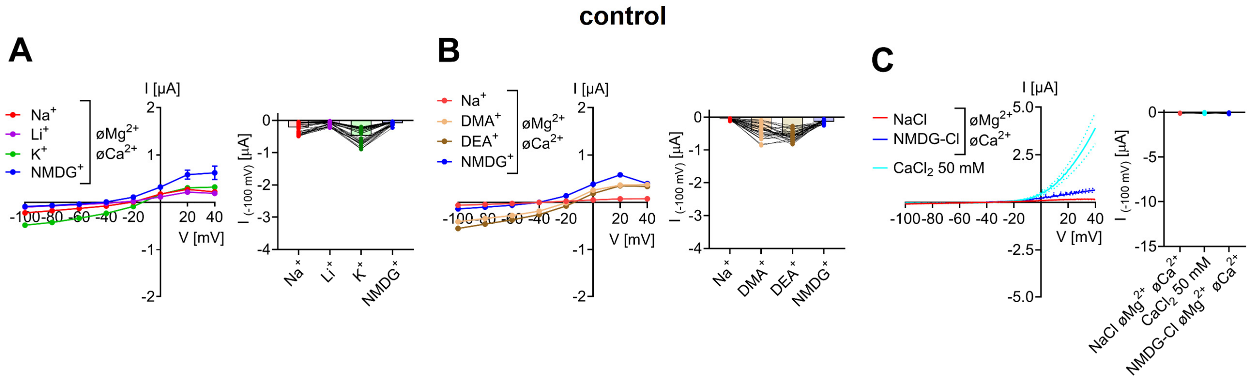

Fig. S9. Effect of monovalent cation substitutions and application of 50 mM $CaCl_2$ bath solution on baseline currents in control oocytes. *A*-*C*, Baseline currents measured in control oocytes injected solely with AS Cx38 using a similar experimental protocol as described is Fig.3. *Left panels* Average I/V-plots (mean ± SEM). *Right panels* The maximal inward currents at -100 mV. Average values and individual data points are shown (***A***, *n* =36, N=3; ***B***, *n* =28, N=3; ***C***, *n* =21, N=3). *n* indicates the number of individual oocytes analyzed per experimental group and N indicates the number of different batches of oocytes. Lines connect data points obtained from one oocyte. In Fig. 3 and Fig.S10, these average whole-cell currents in different bath solutions were used to correct corresponding current values obtained in oocytes expressing polycystin constructs for endogenous oocyte currents.

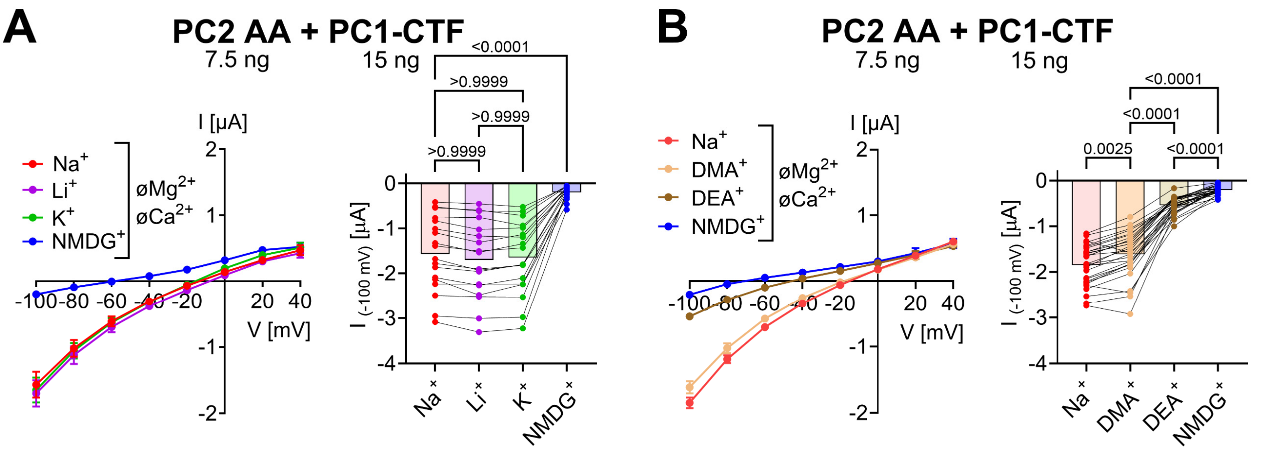

Fig. S10. Permeability for monovalent cations of heteromeric PC2 AA / PC1-CTF ion channels. ***A***, ***B***, Permeability for small inorganic monovalent cations (***A***) and mid-size organic monovalent cations (***B***) was assessed as described in Fig. 3. *Left panels* Average I/V-plots (mean ± SEM). *Right panels* The maximal inward currents at -100 mV. The current values were corrected for endogenous oocyte currents shown in Fig. S9. Average values and individual data points are shown (***A***, *n* =18, N=2; ***B***, *n* =29, N=3). *n* indicates the number of individual oocytes analyzed per experimental group and N indicates the number of different batches of oocytes. Lines connect data points obtained from one oocyte. The *p*-values were calculated by the repeated measures one-way ANOVA with Bonferroni's post hoc test.

PC2 AA + PC1-CTF AAA

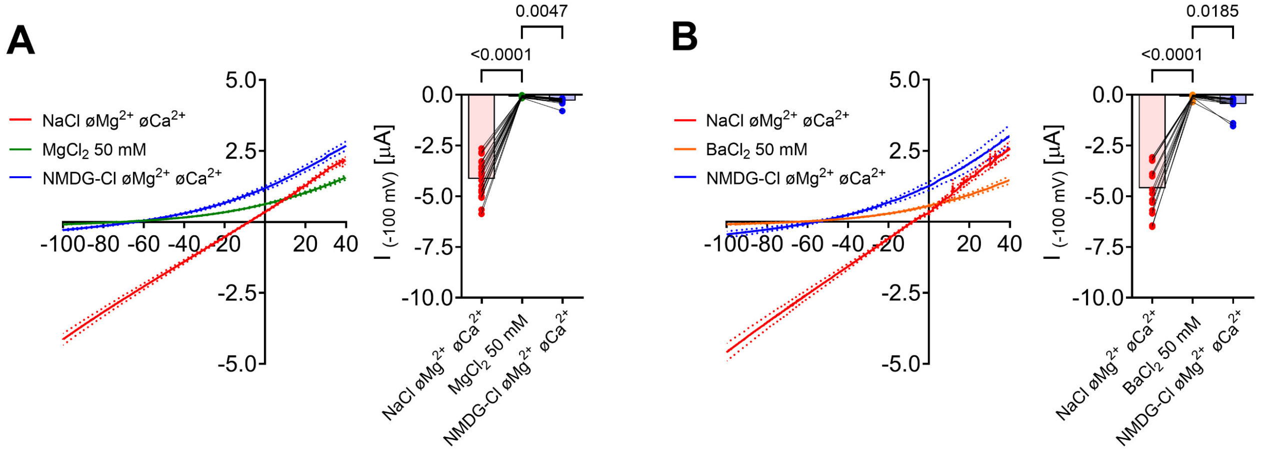

Fig. S11. Heteromeric PC2 AA / PC1-CTF AAA ion channels are impermeable for divalent cations Mg^{2+} and Ba^{2+}. ***A***, ***B*** Permeability for Mg^{2+} (***A***) or Ba^{2+} (***B***) was estimated using a similar experimental protocol as described is Fig.3***E***, ***F*** but with 50 mM $MgCl_2$ or 50 mM $BaCl_2$ bath solutions, respectively. *Left panels* Average I/V-plots (mean ± SEM). *Right panels* Maximal inward currents at -100 mV. Average values and individual data points are shown (***A***, *n* =20, N=2; ***B***, *n* =15, N=2). *n* indicates the number of individual oocytes analyzed per experimental group and N indicates the number of different batches of oocytes. Lines connect data points obtained from one oocyte. The *p*-values were calculated by the Friedman test with Dunn's post hoc test.

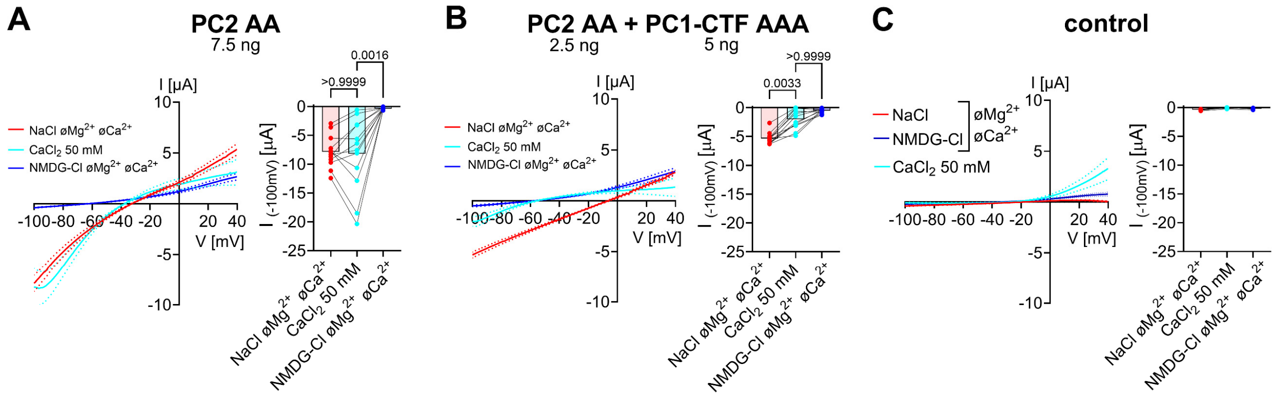

Fig. S12. Heteromeric PC2 AA / PC1-CTF AAA ion channels are less permeable for Ca^{2+} than PC2 AA homomers even under experimental conditions facilitating Ca^{2+} entry. ***A*-*C*** Permeability for Ca^{2+} in oocytes expressing PC2 AA (***A***), co-expressing PC2 AA and PC1-CTF AAA (***B***), and control oocytes (***C***) was estimated using a similar experimental protocol as described is Fig.3***E***, ***F*** but with a continuous holding potential of 0 mV instead of -60 mV between ramp protocols to reduce the voltage-dependent pore blocking effect of Ca^{2+} (see Methods). The current values shown in ***A*** and ***B*** were corrected for endogenous oocyte currents shown in ***C***. *Left panels* Average I/V-plots (mean ± SEM). *Right panels* Maximal inward currents at -100 mV. Average values and individual data points are shown (***A***, *n* =12, N=2; ***B***, *n* =12, N=2; ***C***, *n* =6, N=2). *n* indicates the number of individual oocytes analyzed per experimental group and N indicates the number of different batches of oocytes. Lines connect data points obtained from one oocyte. The *p*-values were calculated by the Friedman test with Dunn's post hoc test.

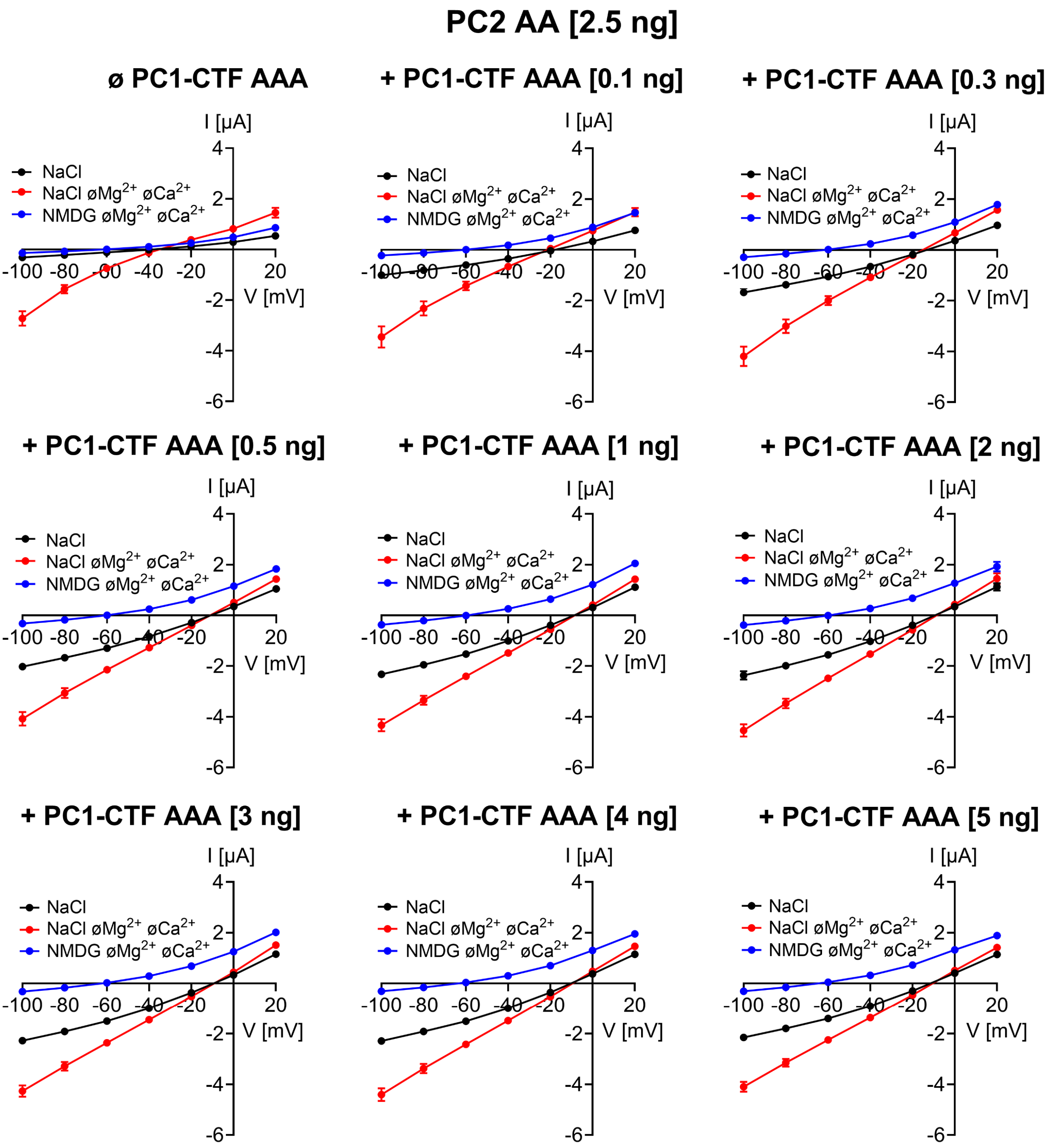

Fig. S13. Average I/V plots from coexpression experiments using a fixed amount of PC2 AA and increasing amounts of PC1-CTF AAA. Average I/V-plots (mean ± SEM; $13 \le n \le 27$, N=2-3) obtained in different bath solutions using the same experimental approach as shown in Fig. 1 from oocytes co-injected with a constant amount of PC2 AA (2.5 ng) and a variable amount of PC1-CTF AAA as indicated. *n* indicates the number of individual oocytes analyzed per experimental group and N indicates the number of different batches of oocytes. Average I/V plots shown in Fig. 4 for NaCl bath solution without divalent cations (NaCl øMg^{2+}øCa^{2+}) are extracted from the I/V plots shown here.

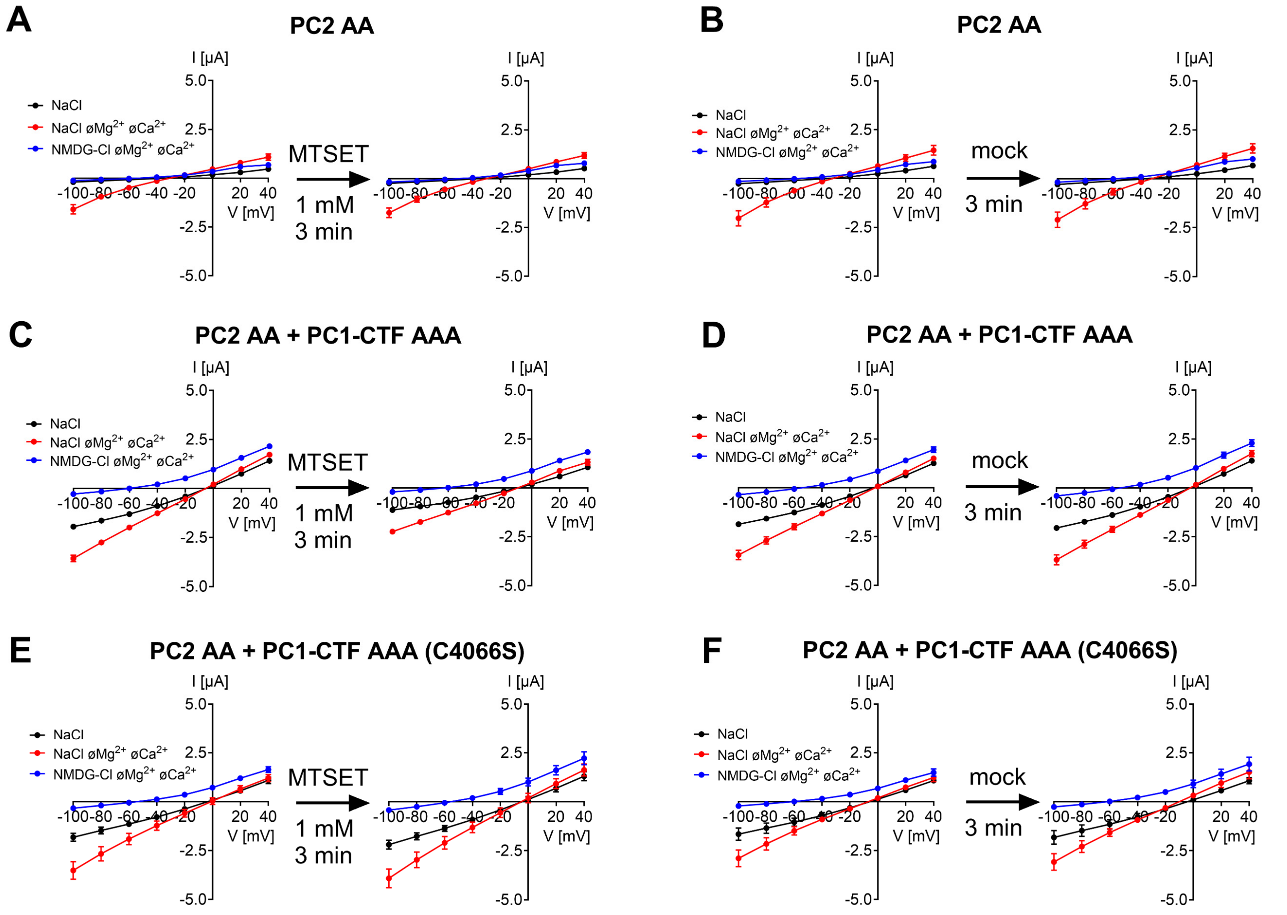

Fig. S14. Sulfhydryl reagent MTSET inhibits PC2 AA / PC1-CTF AAA heteromers through covalent modification of the pore-loop residue C4066. Average I/V-plots (mean ± SEM; 8≤*n*≤12, N=2-3) obtained in different bath solutions using the same experimental approach as shown in Fig. 1 from oocytes expressing PC2 AA alone (***A***, ***B***), co-expressing PC2 AA and PC1-CTF AAA (***C***, ***D***), or co-expressing PC2 AA and PC1-CTF AAA with additional C4066S mutation (***E***, ***F***). *n* indicates the number of individual oocytes analyzed per experimental group and N indicates the number of different batches of oocytes. In each individual oocyte currents were measured before and after 3 min incubation in NaCl bath solution supplemented with 1 mM MTSET (***A***, ***C***, ***E***). The oocyte was unclamped during the incubation time. Before the second current measurement, MTSET was washed out with NaCl bath solution. Impaling microelectrodes were not removed from the oocyte until the end of the experiment. Mock-treated control oocytes were incubated for 3 min in NaCl bath solution without MTSET (***B***, ***D***, ***F***). Average I/V plots shown in Fig. 5B for NaCl bath solution without divalent cations (NaCl øMg^{2+}øCa^{2+}) are extracted from the I/V plots shown here.

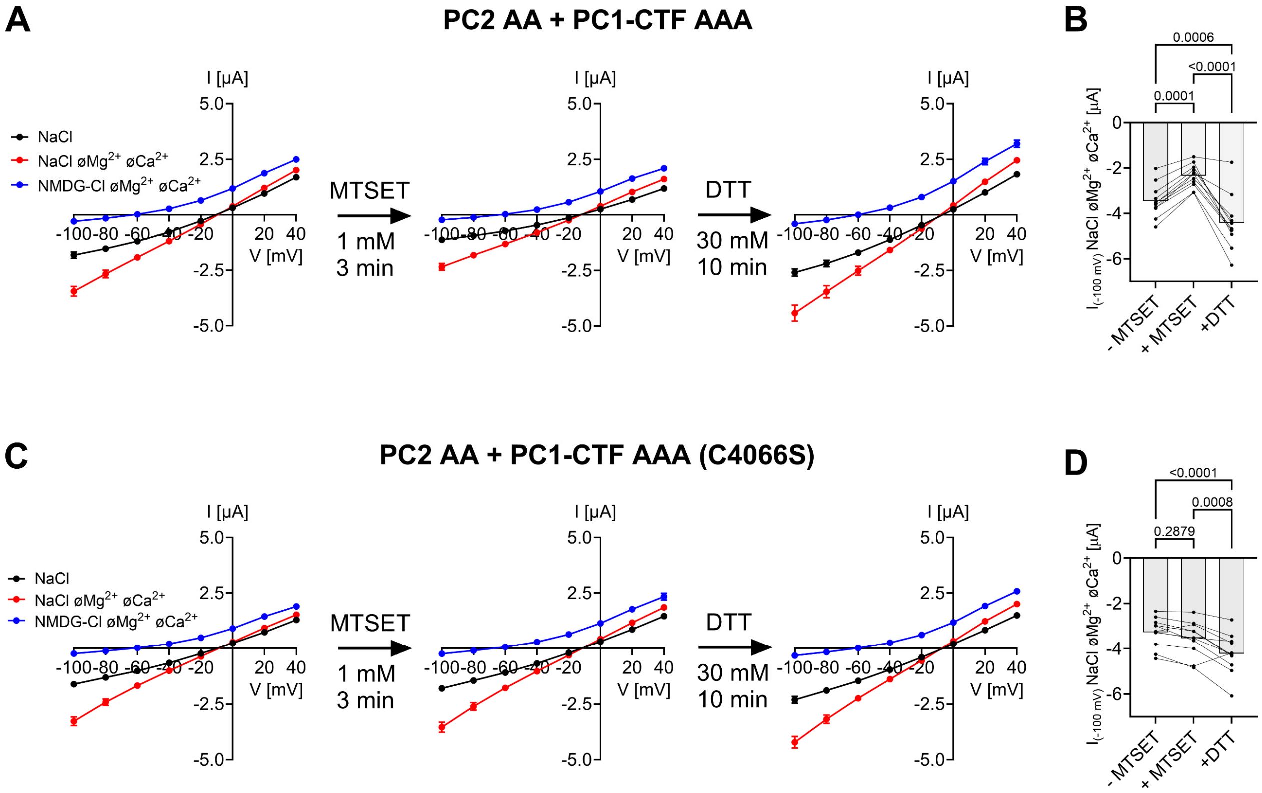

Fig. S15. The inhibitory effect of MTSET on ion channel function of PC2 AA / PC1-CTF AAA heteromers can be rescued by DTT. Average I/V-plots (mean ± SEM; ***A***, ***C***) and the corresponding summary data showing the maximal inward current reached at -100 mV in divalent free NaCl (NaCl øMg^{2+} øCa^{2+}) bath solution (mean values and individual data points; ***B***, ***D***) are presented. In each individual oocyte the currents were measured before the treatment with MTSET (- MTSET), after 5 min incubation in NaCl bath solution supplemented with 1 mM of MTSET (+ MTSET), and after 10 min incubation in NaCl bath solution supplemented with 30 mM of DTT (+ DTT). The experimental protocol was similar to that described for Fig.5. Lines connect data points obtained from one oocyte. It is noteworthy, that the slow spontaneous current run-up observed in oocytes co-expressing PC2 AA and PC1-CTF AAA with the C4066S mutation (***C***, ***D***) was clearly different from the acute inhibitory response to MTSET and the subsequent rescue effect of DTT observed in oocytes co-expressing PC2 AA and PC1-CTF AAA (***A***, ***B***). The *p*-values were calculated by the repeated measures one-way ANOVA with Bonferroni's post hoc test (*n* =11, N=2). *n* indicates the number of individual oocytes analyzed per experimental group and N indicates the number of different batches of oocytes.

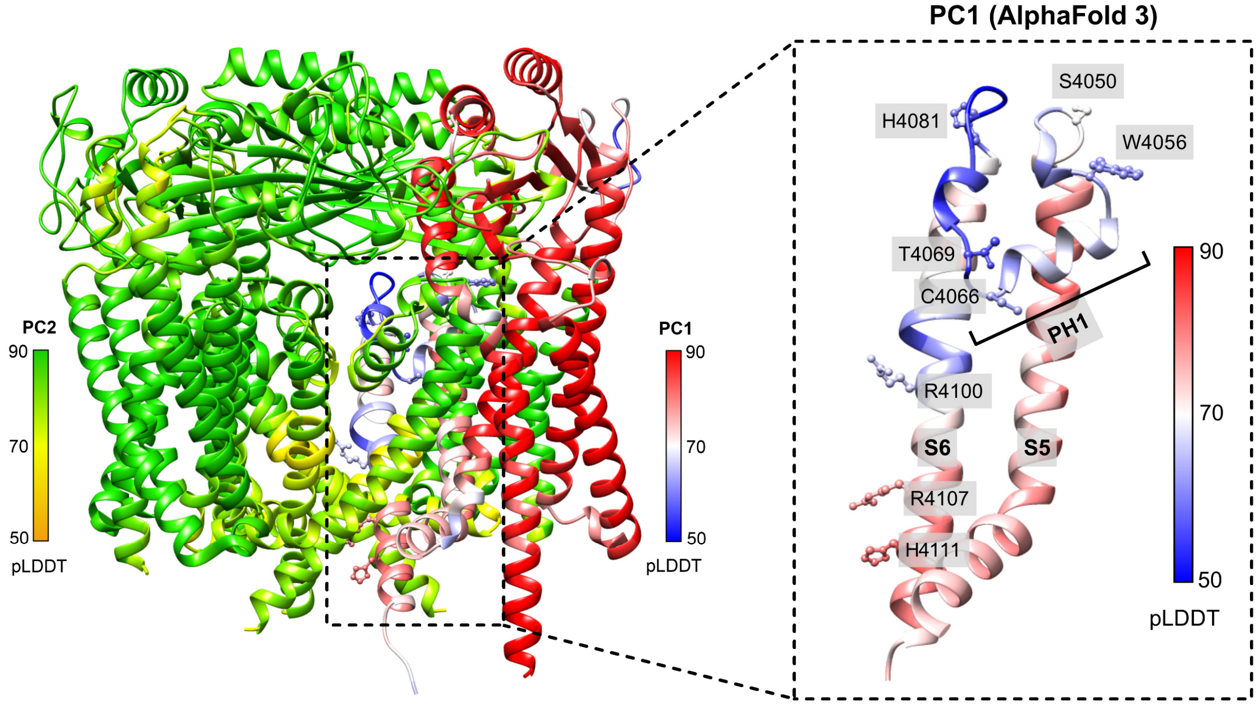

Fig. S16. AlphaFold 3-generated model of the PC2/PC1 heterocomplex. Side view of the PC2/PC1 heterotetramer model, depicted as ribbon diagrams and coloured by pLDDT – a confidence estimate on a 0-100 scale, where a higher value indicates higher confidence. PC2 subunits are shown in orange-yellow-green, PC1 subunit is shown in blue-white-red. The high-confidence AlphaFold 3 model of the PC2/PC1 heteromer was obtained (confidence score: 0.8413; pTM scores: PC1: 0.9172, PC2: 0.9351, 0.9241, 0.935). The structure of the PC1 pore loop region was predicted with lower confidence, with pLDDT values averaging 63.19 and 70.06 for the proximal (W4056-T4069) and distal (H4081-V4088) portions of the pore loop, respectively. Nevertheless, the AlphaFold 3 model supported the canonical TRP-like pore loop conformation of PC1.

Table S1. Reagents and Tools.

Reagent/Resource	Reference or Source	Identifier or Catalog Number
**Experimental Models**		
Xenopus laevis oocytes	Nasco Education, Fort Atkinson, USA	N/A
**Recombinant DNA**		
pTLN vector	Lorenz *et al.*, 1996 [1]	N/A
human polycystin-2	R. Witzgall (Regensburg, Germany)<br>Staudner *et al.*, 2024 [2]	N/A
human polycystin-1	R. Witzgall (Regensburg, Germany); modified in this study	N/A
**Antibodies**		
rat-anti-HA (monoclonal)	Roche Diagnostics	clone 3F10<br>Ref: 11867431001<br>Lot: 13565000
mouse-anti-V5 (monoclonal)	Thermo Fisher Scientific	Ref: R96025<br>Lot: 2735895
rabbit-anti-β-actin (polyclonal)	Sigma Aldrich	Ref: A2066<br>Lot: 086K4855
goat-anti-rat (polyclonal)	Jackson Immunoresearch	Ref: 112-035-006<br>Lot: 111926
goat-anti-mouse (polyclonal)	Abcam	Ref: ab97023<br>Lot: GR3242292-6
goat-anti-rabbit (polyclonal)	Invitrogen	Ref: G21234<br>Lot: 2273627
**Oligonucleotides and other sequence-based reagents**		
antisense phosphorothioate oligoDNA against *X. laevis* connexin 38 (AS Cx38):<br>5'-GCT TTA GTA ATT CCC ATC CTG CCA TGT TTC-3'	biomers.net	N/A
**Chemicals, Enzymes and other reagents**		
mMessage mMachine SP6	Invitrogen by Thermo Fisher Scientific	Ref: AM1340<br>Lot: ZC382878
QuikChange<br>Lightning site-directed mutagenesis kit	Agilent	Ref: 210518/210519
Collagenase type-2 from Clostridium hystolyticum	Sigma Aldrich	Ref: C6885
Thermo Scientific™ SuperSignal™ West Femto Maximum Sensitivity Substrate	Thermo Fisher Scientific	Ref: 34096<br>Lot: ZC382878
EZ-Link™ Sulfo-NHS-SS-Biotin	Thermo Fisher Scientific	Ref: 21331

		Lot: ZE388820
cOmplete™ EDTA-free Protease Inhibitor Cocktail	Roche Diagnostics	Ref: 04 693 132 001<br>Lot: 59148200
Pierce Neutravidin Agarose	Thermo Fisher Scientific	Ref: 29201<br>Lot: YF362925
Pierce Anti-HA magnetic beads	Thermo Fisher Scientific	Ref: 88836<br>Lot: XD335925
Anti-V5 magnetic beads	Sigma Aldrich	Ref: SAE0203<br>Lot: 0000205518
[2-(Trimethylammonium) ethyl] methanethiosulfonate Bromide, MTSET	Toronto Research Chemicals, TRC	Ref: TRC-T795900<br>Lot: 10-RFS-55-4
DL-Dithiothreitol, DTT	Sigma Aldrich	Ref: D9779<br>Lot: SLCJ5918
**Software**		
GraphPad Prism v. 10.2.1	https://www.graphpad.com	N/A
Microsoft Excel 2021	https://www.microsoft.com/	N/A
UCSF Chimera	https://www.cgl.ucsf.edu/chimera/<br>Pettersen et al., 2004 [3]	N/A
CLUSTAL Omega	https://www.uniprot.org/align<br>http://www.clustal.org/omega	N/A
MODELLER	https://salilab.org/modeller/	N/A
REFMAC	https://www2.mrc-lmb.cam.ac.uk/groups/murshudov/content/refmac/refmac.html	N/A
COOT	https://www2.mrc-lmb.cam.ac.uk/personal/pemsley/coot/	N/A
Nest-o-patch	https://sourceforge.net/projects/nestopatch/<br>Viatcheslav V. Nesterov	N/A
Pulse v8.78	HEKA Elektronik Dr. Schulze GmbH, Lambrecht, Germany	N/A
**Other**		
Nanoject II microinjector	Drummond Scientific, Broomall, USA	N/A
P-97 microelectrode puller	Sutter Instrument, Novato, USA	N/A
OC-725C oocyte clamp amplifier	Warner Instruments Corp., Hamden, USA	N/A
LIH 1600 acquisition interface	HEKA	N/A
Valve Commander $VC^3 8$ System	ALA scientific instruments Inc., Farmingdale, USA	N/A
ALA VM8 perfusion system	ALA scientific instruments Inc., Farmingdale, USA	N/A

Table S2. Comparison of the model-*versus*-data metrics for PC1/PC2 heterocomplex of the published (PDB ID: 6A70) and re-interpreted model (this study).

Metric	6A70.pdb[a]	this study[b]
no. residues	1786	1838
no. atoms	13237	15008
MolProbability score[1]	1.84	1.77
CaBLAM outliers[2]	4.75 %	3.22 %
ADP (B-factors)[3] min/max/mean	0.00/215.33/90.42	36.97/160.00/115.76
CC_{box}[4]	0.52	0.59
CC_{mask}[5]	0.59	0.74
CC_{volume}[6]	0.58	0.73
CC_{peaks}[7]	0.34	0.43

For the comparison, the N-terminal domain (NTD, res. 3075-3657) from the original model was omitted in both models. Metrics were prepared using Python-based Hierarchical ENvironment for Integrated Xtallography [4].

[a] Model residues of PC1: 3657-3752, 3783-4050, 4081-4120; PC2 (monomer 1): 219-291, 313-463, 471-694; PC2 (monomer 2): 219-293, 313-699; PC2 (monomer 3): 219-294, 305-699.
[b] Model residues of PC1: 3657-3749, 3783-3820, 3826-4122; PC2 (monomer 1): 216-294, 306-694; PC2 (monomer 2): 216-295, 304-694; PC2 (monomer 3): 216-295, 304-694.

[1]A log-weighted composite value of the clash-score, percentage Ramachandran not favored and percentage bad sidechain rotamers (lower = better).
[2] System to evaluate protein CA mainchain geometry in low-resolution structures.
[3] Atomic displacement parameter (ADP), uncertainty in atomic positions.
[4] Correlation coefficient, similarity of model and target maps.
[5] Correlation coefficient, fit of the atomic centers.
[6] Correlation coefficient, fit of the molecular envelope defined by the model map.
[7] Correlation coefficient, fit of the strongest peaks in the model and target maps.

Supporting Information References

1. Lorenz, C., Pusch, M. & Jentsch, T. J. (1996) Heteromultimeric CLC chloride channels with novel properties. *Proc. Natl. Acad. Sci. U S A.* 93, 13362-13366.

2. Staudner, T., Geiges, L., Khamseekaew, J., Sure, F., Korbmacher, C. & Ilyaskin, A. V. (2024) Disease-associated missense mutations in the pore loop of polycystin-2 alter its ion channel function in a heterologous expression system, *J Biol Chem*. 300, 107574.

3. Pettersen, E. F., Goddard, T. D., Huang, C. C., Couch, G. S., Greenblatt, D. M., Meng, E. C. & Ferrin, T. E. (2004) UCSF Chimera--a visualization system for exploratory research and analysis, *J Comput Chem*. 25, 1605-1612.

4. Liebschner, D., Afonine, P. V., Baker, M. L., Bunkóczi, G., Chen, V. B., Croll, T. I., Hintze, B., Hung, L. W., Jain, S., McCoy, A. J., Moriarty, N. W., Oeffner, R. D., Poon, B. K., Prisant, M. G., Read, R. J., Richardson, J. S., Richardson, D. C., Sammito, M. D., Sobolev, O. V., Stockwell, D. H., Terwilliger, T. C., Urzhumtsev, A. G., Videau, L. L., Williams, C. J. & Adams, P.D. (2019) Macromolecular structure determination using X-rays, neutrons and electrons: recent developments in Phenix. *Acta Crystallogr. D. Struct. Biol.* 75, 861-877.
